# ’s Copper-FomA Proteins: Driving Cancer Forward

**DOI:** 10.1021/acs.inorgchem.5c01747

**Published:** 2025-07-19

**Authors:** Monika K. Lesiów, Bartosz Kwiatkowski, Piotr Pietrzyk, Agnieszka Kyzioł, Krzysztof Rolka, Urszula K. Komarnicka

**Affiliations:** † Faculty of Chemistry, 49572 University of Wrocław, F. Joliot-Curie 14, 50-383 Wrocław, Poland; ‡ Faculty of Chemistry, 37799 Jagiellonian University, Gronostajowa 2, 30-387 Kraków, Poland; § Faculty of Chemistry, 49646 University of Gdańsk, Wita Stwosza 63, 80-308 Gdańsk, Poland

## Abstract

FomA is a major outer membrane protein of (Fn) overexpressed in colorectal cancer (CRC) tissues, where increased levels of Cu ions together with reactive oxygen species (ROS) were noted. In this comparative study, the connection between *Fn* surrounded by copper ions and the ROS overgeneration leading to the development of CRC is examined. Our study focused on a model peptide Ac-KGHGNGEEGTPTVHNEYH-NH_2_ (**6L**) derived from the FomA reflecting a fragment of the protein loop no. 4, exposed to the external environment, which facilitates the binding of Cu ions. The coordination studies (potentiometric titration, UV–Vis, CD, EPR) showed that the **6L** bound Cu­(II) and formed mono-, di-, and trinuclear complexes depending on the solution pH. The ability to generate ROS by promoting the Cu­(III)/Cu­(II)/Cu­(I) redox cycle was proven by CV and EPR techniques. We also confirmed that upon adding Asc, the **6L** was fragmented and after copper coordination, the ROS production increased (e.g.,^•^OH, ^1^O_2_, O_2_^•‑^), leading to DNA damage. Stimulation of model mouse colon carcinoma cells by Cu­(II) complex with **6L** demonstrated an abundant cellular ROS production, resulting in pronounced lipid peroxidation. Hypothetically, this action mode leads to the damage of colon cells and triggers carcinogenesis processes.

## Graphical abstract

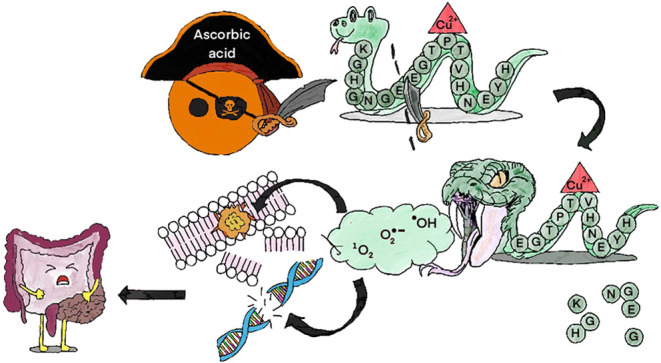

## Introduction

Copper ions coordination processes to various peptides are intensively studied in the literature, especially those associated with developing oxidative stress-related neurodegenerative diseases.^1–8^ The most studied systems of Cu­(II)-peptide complexes related to such disorders are those containing fragments of amyloid-β peptide (Alzheimer’s disease),^9–11^ Tau protein (microtubule-associated protein),^12^ presenilin 1 (Prs1) (a component of γ-secretase complex),^13^ prion (PrPC) (a cell-surface glycoprotein),^14^ and α-synuclein (a neuronal protein).^15^ In addition to neurodegenerative diseases, Cu­(II) peptide complexes may participate in developing other pathogenic conditions. Spike protein fragments from SARS-CoV-2 virus can bind Cu­(II) ions and efficiently generate hydroxyl radical and superoxide anion radicals, which damage DNA.^16^ Moreover, these complexes generate mitochondrial ROS in the lung cells and disrupt the Cu­(I) level in the mitochondria, which may be associated with the development of COVID-19.^17^

However, as is widely known, ROS generation may also influence the development of other diseases, such as cancer. ROS led to double-strand DNA breaks and cause 8-oxo-7-hydro-2′-deoxyguanosine formation (8-oxodG), the accumulation of which is responsible for the initiation of various cancer types.^18,19^ In our studies, we decided to focus on participation of ROS in colorectal cancer (CRC) initiation. This idea was prompted by literature data indicating elevated levels of copper ions and ROS in the tissues of patients with CRC.^20,21^ Furthermore, it has been suggested that (Fn) may have a major impact on the occurrence of colorectal cancer.^22^ Taking this as a motivation, we took into consideration a few fragments (Ac-KGHGNG-NH_2_ (**1L**),^23^ Ac-PTVHNE-NH_2_ (**2L**),^23^ Ac-KGHGNGEEGTPTVHNE-NH_2_ (**3L**)^24^ and its cyclic counterpart, cyclo­(KGHGNGEEGTPTVHNE) (**4L**)),^24^ as well as Ac-PTVHNEYH-NH_2_ (**5L**)^25^ from loop no.4 (reach in His residues) of the FomA protein from *Fn* and analyzed their interaction with Cu­(II) ions. Our results showed that the peptide fragments bound Cu­(II) ions and upon complex formation in the presence of H_2_O_2_, Asc, or their mixture generated reactive oxygen species (ROS).^23,25^ In addition to DNA damage, ROS caused lipid peroxidation, leading to colorectal cancer development.^26,27^

Our previous studies provided extensive information on ROS production and its potential role in cancer initiation. However, we aimed to take it a step further by investigating how ROS production activity would change together with elongation of peptide fragments containing all of the previously studied fragments. Such a long peptide fragment brings us much closer to the real situation in the body, when all possible metal ion binding sites are available in loop no. 4 of the FomA protein. Thus, possible binding of more than one Cu­(II) ion may affect the activity toward generating ROS by the formed complex. It seems interesting to investigate the difference in the level of ROS generated by the complex with the newly designed fragment as well as the efficiency of damaging biomolecules by the generated ROS compared to previously studied systems with FomA protein fragments.

Thus, in this article, we decided to focus on a model FomA peptide fragment consisting of 18 amino acid residues (Ac-KGHGNGEEGTPTVHNEYH-NH_2_) (**6L**), which constitutes more than half of loop no. 4 of the FomA protein and contains three histidyl residues—the most important binding sites for Cu­(II) ions present in the loop (Figure 1). Figure 1 shows the fragments of the FomA protein that we studied previously (Ac-KGHGNG-NH_2_ (**1L**),^23^ Ac-PTVHNE-NH_2_ (**2L**),^23^ Ac-KGHGNGEEGTPTVHNE-NH_2_ (**3L**),^24^ cyclo­(KGHGNGEEGTPTVHNE) (**4L**),^24^ as well as Ac-PTVHNEYH-NH_2_ (**5L**)^25^) and that are part of the newly studied in this work **6L** peptide.

**Figure 1:**
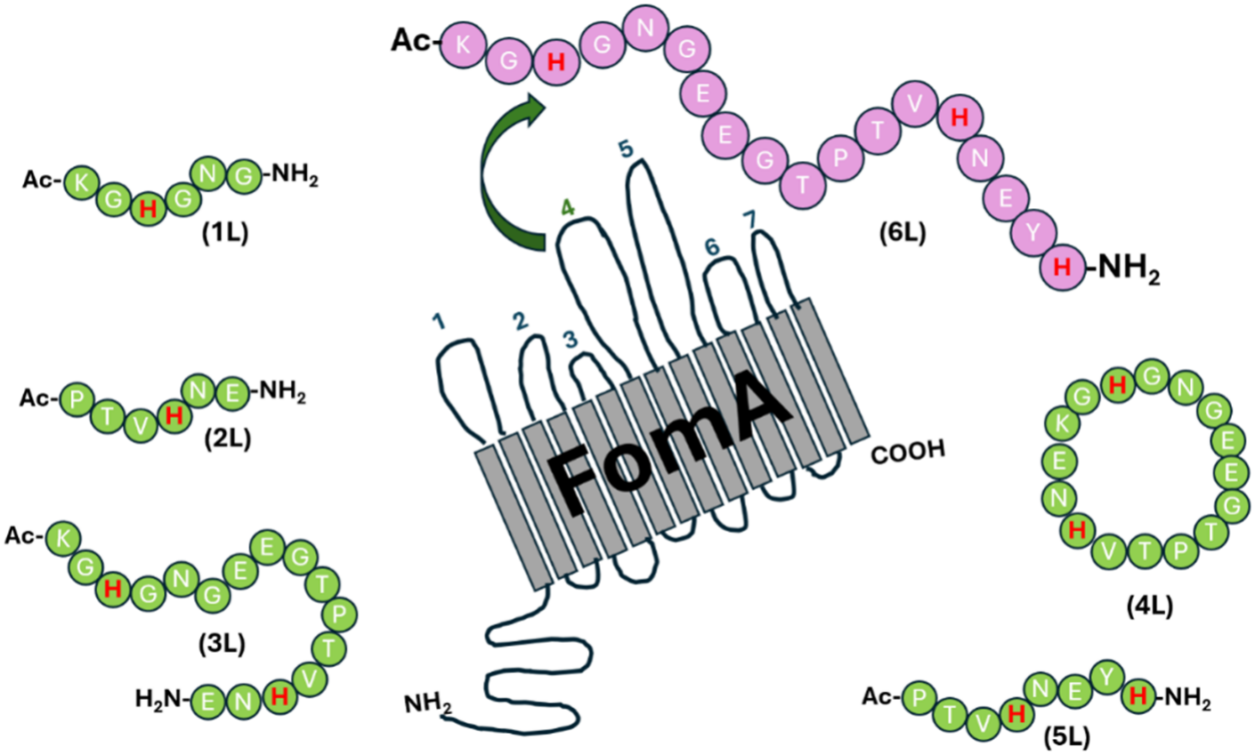
Schematic representation of the FomA protein of the with peptide fragments studied by us previously and the newly designed **6L** ligand.

## Results and Discussion

### Characterization of the Cu­(II)-Ac-KGHGNGEEGTPTVHNEYH-NH_2_ (Cu6L) System

The biological activity of the system depends on the structure of the complex that is formed under given conditions.^28^ Therefore, our studies have focused from the beginning on determining the coordination mode of the complex mainly in the pH of the large intestine (pH 6.8) but also in the entire pH range. However, here we will focus on the characteristics of the systems that dominate in the intestinal environment, and the description of the complexes formed in the remaining pH range from 2.5 to 10.5 is presented in the Supporting Information (Tables S1–S5).

At the pH of the large intestine (6.8) in a 1:1 M:L molar ratio, two species (CuH_2_L and CuHL) predominate (Figure 2A). The deprotonation of the 2N complex and formation of the CuH_2_L species (about 65% of the solution at pH 6.2) are observed above pH 4.0. The p*K*_a(3/2)_ value of the CuH_3_L ↔ CuH_2_L + H^+^ reaction equals 5.65 and may be related to the deprotonation and coordination of the imidazole nitrogen atom from the histidyl residue to the Cu­(II) ion (Table S2).^29^ The calculated log K* value (−10.96), EPR parameters (*A*_||_ = 17.0 mT, *g*_||_ = 2.282), and the hypsochromic shift of the d–d transition band in the UV–Vis spectrum from 671 to 635 nm (the value calculated by the Prenesti method is 634 nm) suggest the involvement of the third imidazole nitrogen atom in the Cu­(II) ion binding (Table 1 and Figures S1B and 2B).^30,31^ The stability of the CuH_2_L complex is comparable to those reported in the literature for Cu­(II)-Ac-HAHVH-NH_2_ and Cu­(II)-Ac-HLHWH-NH_2_, also containing three His residues in the peptide sequence and representing the same 3N {3N_im_} coordination mode.^32^

**Figure 2:**
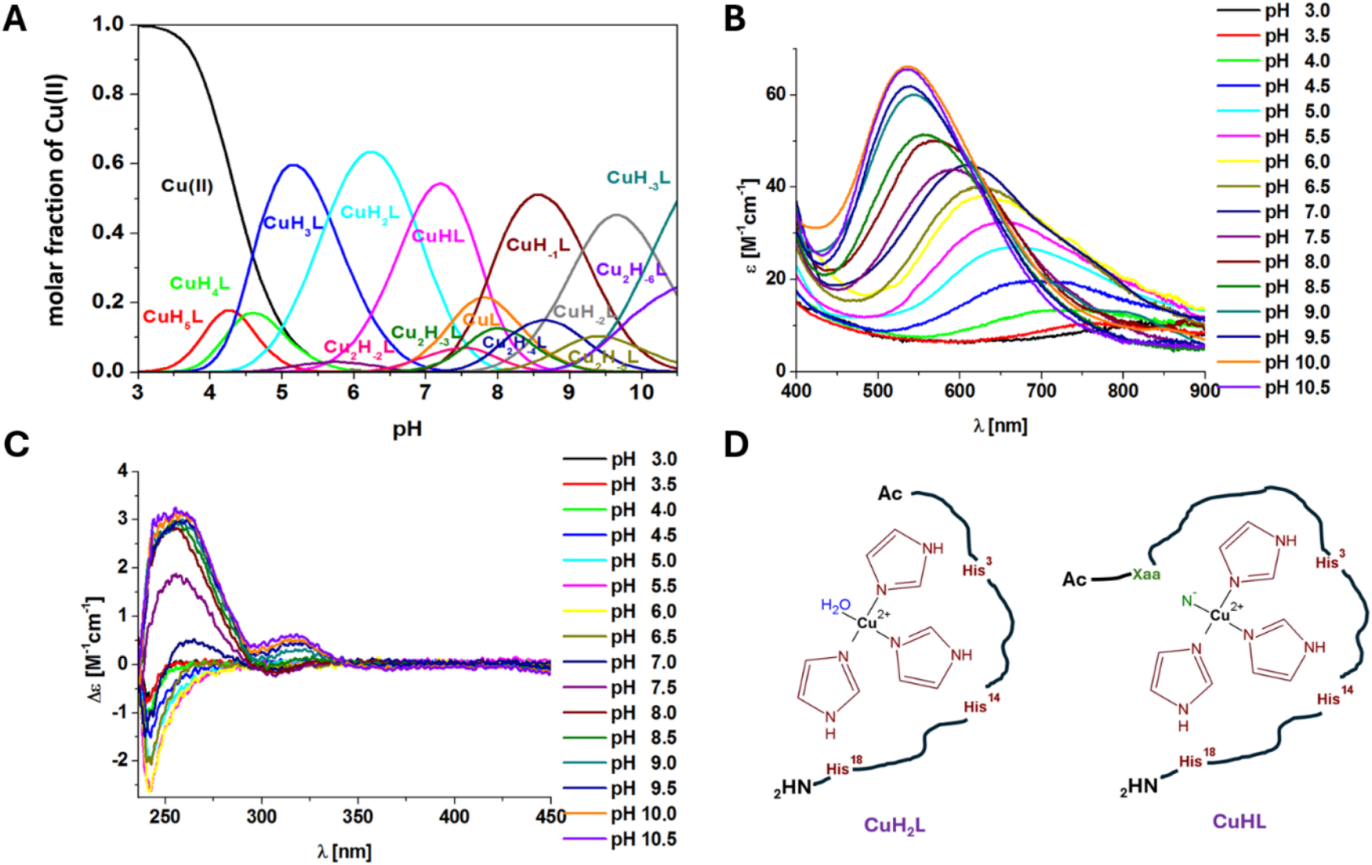
Coordination studies carried out for the Cu­(II)–Ac-KGHGNGEEGTPTVHNEYH-NH_2_ (**Cu6L**) system: (A) species distribution diagram of the complex as a function of pH (1:1 M:L molar ratio, [Cu­(II)] = 0.001 M); (B) electronic absorption spectra of the complex as a function of pH (1:1 M:L molar ratio, [Cu­(II)] = 0.001 M); (C) CD spectra of the complex in the UV region as a function of pH (1:1 M:L molar ratio, [Cu­(II)] = 0.001 M); (D) schematic representation of the CuH_2_L and CuHL structures of the **Cu6L** system.

**Table 1:** Calculated Values of Log K* for the Mononuclear Cu­(II) Complexes with FomA Protein Fragments (Ac-KGHGNG-NH_2_ (**1L**), Ac-PTVHNE-NH_2_ (**2L**), Ac-KGHGNGEEGTPTVHNE-NH_2_ (**3L**), Cyclo­(KGHGNGEEGTPTVHNE) (**4L**), Ac-PTVHNEYH-NH_2_ (**5L**), and Ac-KGHGNGEEGTPTVHNEYH-NH_2_ (**6L**))

	Coordination mode						
	2N^b^{2N_im_}	3N{N_im_,2N^–^}	3N{2N_im_,N^–^}	3N{3N_im_}	4N{2N_im_,2N^–^}	4N{3N_im_,N^–^}	4N{N_im_,3N^–^}
system	log K*^a^						
**Cu1L** ^c^	-	–14.95	-	-	-	-	–20.31
**Cu2L** ^c^	-	–14.94	-	-	-	-	–21.95
**Cu3L** ^d^	–6.09	-	–12.87	-	-	-	–21.31
**Cu4L** ^d^	–5.78	-	–12.49	-	-	-	–21.03
**Cu5L** ^e^	-	-	–12.11	-	–18.99	-	–23.85
**Cu6L**	–5.31	-	-	–10.96	-	–17.78	–20.80

a logK* values are given for the reaction: Cu^2+^+H*_n_* L ⇄ MH*_j_* L^(2– *n*+*j*)+^ + (*n* – *j*)­H^+^; logK* = log β­(CuH*_j_* L) – log β­(H*_n_* L) (where the index *j* corresponds to the number of protons in the coordinated ligand to metal ion and *n* corresponds to the number of protons of the coordinated ligand and release from ligand during complexation).

b 2N, 3N, or 4N indicates the number of nitrogen atoms directly coordinated to copper­(II) ion, whereas N_im_ and N^–^ indicate nitrogen atoms of imidazole and amide groups, respectively.

c^23^

d^24^

e^25^

By increasing the pH value, the first amide nitrogen atom is deprotonated, and a new CuHL species (with p*K*_a(2/1)_ = 6.82) is formed (Figure 2A and Table S2).^33^ In the UV–Vis spectrum, a hypsochromic shift of the d-d band from 635 to 591 nm is observed (the value obtained from the Prenesti equation is 563 nm) (Figure 2B).^34^ The difference between the experimental and theoretical wavelengths may result from additional ligand interactions with the metal ion. The bands at 256, 307, and 347 nm in the CD spectrum can be assigned to the following charge transfer transitions: N_Im_π_2_ → Cu­(II), N_(amide)_^–^ → Cu­(II), and N_Im_π_1_ → Cu­(II), respectively (Table S3 and Figure 2C).^35,36^ The parameters obtained from the EPR spectrum for the CuHL complex are *A*_||_ = 17.4 mT, *g*_||_ = 2.267 (Figure S1C). The obtained spectroscopic parameters for the CuHL species confirm the 4N {3N_im_, N^–^} coordination mode (Figure 2D and Table S3).

Binding two Cu­(II) ions at pH 6.8 (2:1 M:L molar ratio) results in the formation of a dinuclear Cu_2_H_–2_L species (Figure S2 and Table S4). Based on the obtained spectroscopic data: d–d band at 595 nm in the UV–Vis spectrum, CT transitions in the ultraviolet range of the CD spectrum (N_Im_π_2_ → Cu­(II) at 252 nm, N_(amide)_^–^ → Cu­(II) at 307 nm, N_Im_π_1_ → Cu­(II) at 357 nm) and EPR parameters (*A*_||_ = 17.2 mT, *g*_||_ = 2.230), the following 3N {N_im_, 2N^–^} 4N {2N_im_, 2N^–^} or 4N {N_im_, 3N^–^} 3N {2N_im_, N^–^} coordination modes were proposed for the analyzed complex (Figures S3, S4, and S5A, Table S3). The spectroscopic parameters obtained are consistent with the literature for this type of coordination modes.^30,35–37^

In turn, the dominant trinuclear complex in the large intestine environment is Cu_3_H_–4_L (p*K*_a(−3/‑4_) = 6.57) (Table S5 and Figure S6). The obtained p*K*_a_ value confirms the deprotonation and coordination of the sixth amide nitrogen atom.^38^ However, the spectroscopic characterization of this complex is not possible due to the coexistence of the Cu_3_H_–4_L species with other species present in the solution (Figure S6). However, based on the stoichiometry of the Cu_3_H_–4_L species, the 3N {N_im_, 2N^–^} 3N {N_im_, 2N^–^} 3N {N_im_, 2N^–^} donor set is suggested.

Unfortunately, the stability of these complexes formed at the pH of the large intestine (in any M:L molar ratio) cannot be compared with the stability of other Cu­(II) systems with FomA protein fragments (Ac-KGHGNG-NH_2_, **1L**; Ac-PTVHNE-NH_2_, **2L**; Ac-KGHGNGEEGTPTVHNE-NH_2_, **3L**; cyclo­(KGHGNGEEGTPTVHNE), **4L**; Ac-PTVHNEYH-NH_2_, **5L**) that we studied previously because the resulting coordination modes are different (Table 1).

### Peptide Fragmentation

We decided to check the peptide oxidation and fragmentation in the presence of ascorbic acid (Asc). As reported in the literature, Asc participates in metal-ion-catalyzed peptide oxidation, but depending on the studied system, Asc can promote or inhibit ligand oxidation and fragmentation. Data found in the literature show that in the case of the Cu­(II)-Ac-KTDHGA-NH_2_ complex, adding Asc promotes peptide oxidation, while its fragmentation is less favored.^39^ On the other hand, in the case of the Cu­(II)-nMKHA complex reaction with Asc, a protective role of vitamin C and slight peptide oxidation were observed.^40^

Therefore, ESI-MS spectra for **Cu6L** before the addition of Asc and after 1 and 24 h of incubation with Asc were measured. A weakly intense peak of the doubly charged fragment ion [y_12_ + H]^2+^ at 641.794 *m*/*z* was identified after an hour of incubation with Asc, which gained in intensity after 24 h of incubation (Figure 3). The signal of this ion comes from the EGTPTVHNEYH fragment of the Ac-KGHGNGEEGTPTVHNEYH-NH_2_ peptide. This fragment differs only in three amino acid residues from that of the previously studied peptide (Ac-PTVHNEYH-NH_2_, **5L**). These residues are not metal ion binding sites, meaning they do not affect the structure of the formed complex and most likely do not play a significant role in the activity of the complex toward ROS generation. Moreover, the detected peak is not visible in the spectrum of the complex itself, confirming that it is formed just after adding Asc. Another ion that can also be found in the ESI-MS spectrum after 1 and 24 h of incubation of peptide with Asc is [y_11_ + H]^2+^ at 577.273 *m*/*z*. This fragment lacks glutamic acid residue compared to the previously found fragment, but both contain the PTVHNEYH sequence. The obtained results suggest that after adding Asc to the **Cu6L** system, the peptide undergoes fragmentation, and it can have similar activity and ROS-reactivity as the Cu­(II)-PTVHNEYH-NH_2_ (**Cu5L**) complex, which is discussed below.

**Figure 3:**
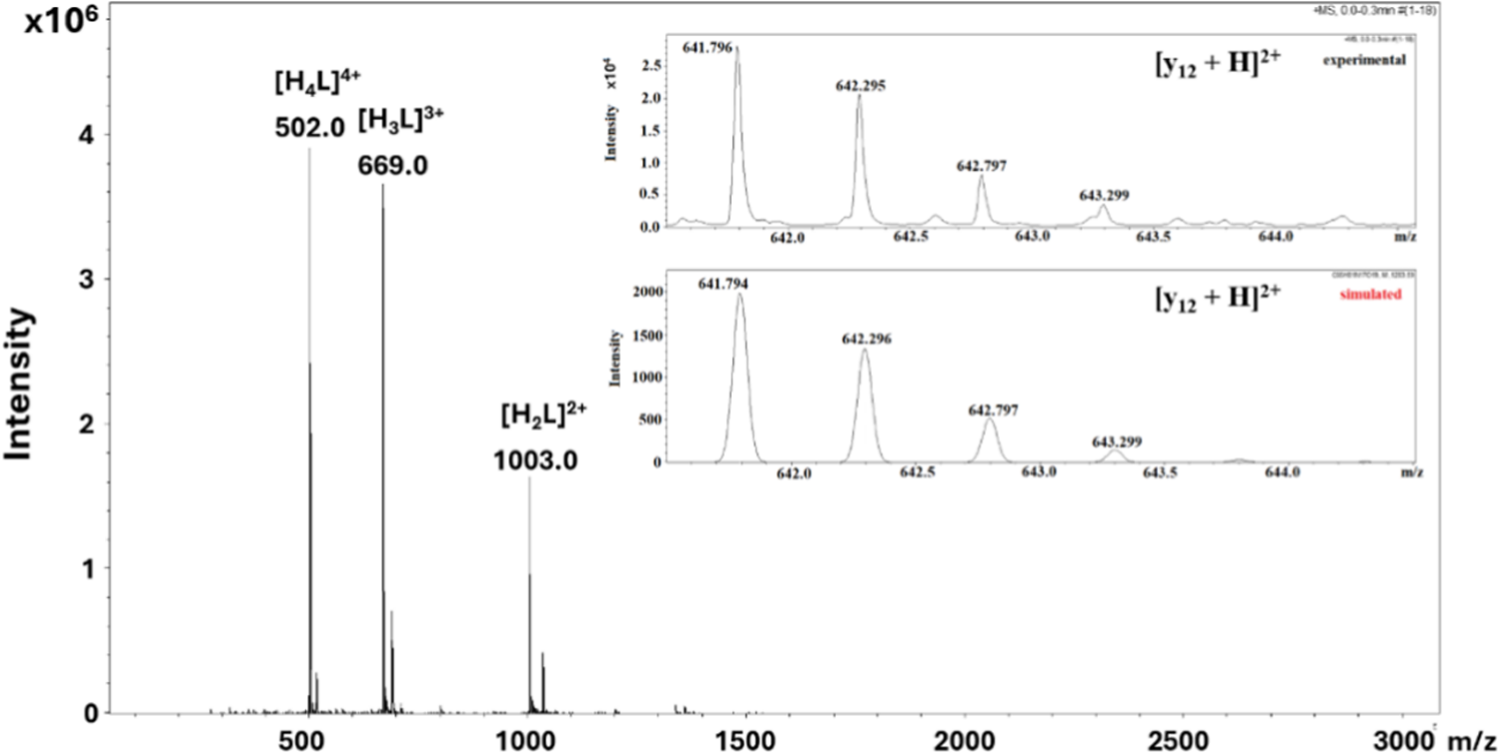
ESI mass spectrum of **Cu6L** system with Asc after 24 h of incubation (1:1 M:L molar ratio, [Cu­(II)] = 1 mM, [Asc] = 1 mM) in aqueous solution. Experimental and simulated spectra of the [y_12_ + H]^2+^ ion (*m*/*z* = 641.794 Da) are shown as an insert.

### Redox Properties of Cu­(II)-FomA Fragments

The redox properties of the **5L** and **6L** ligands, as well as their Cu­(II) complexes (**Cu5L** and **Cu6L**), were also investigated by using cyclic voltammetry. This enabled us to elucidate what happens to the copper ion within the complex during reactive oxygen species (ROS) generation.

Both ligands show one irreversible anodic peak at ∼0.5 V, corresponding to the oxidation of the Tyr amino acid residue present in **5L** and **6L** ligands and the formation of tyrosyl radical (Figure 4A1,B1).^41,42^ By analyzing CV profiles for Cu­(II) complexes, we can observe an anodic signal, most likely the result of several processes (Figure 4A2,B2). The peak at lower potentials is the result of the Cu­(I)/Cu­(II) oxidation process (Table 2).^43^ In turn, the peaks at higher potentials (0.496 and 0.450 V for **Cu5L** and **Cu6L**, respectively) are the additive effect of two phenomena: tyrosine oxidation overlapping with the Cu­(II)/Cu­(III) oxidation process (Table 2 and Figure 4A2,B2).^44,45^ We can observe an increase in this peak for the Cu6L system compared with its **6L** ligand. Moreover, the values of the anodic potentials we obtained for the Cu­(II)/Cu­(III) reaction are slightly lower compared to those reported for other Cu­(II) peptide complexes found in the literature.

**Figure 4:**
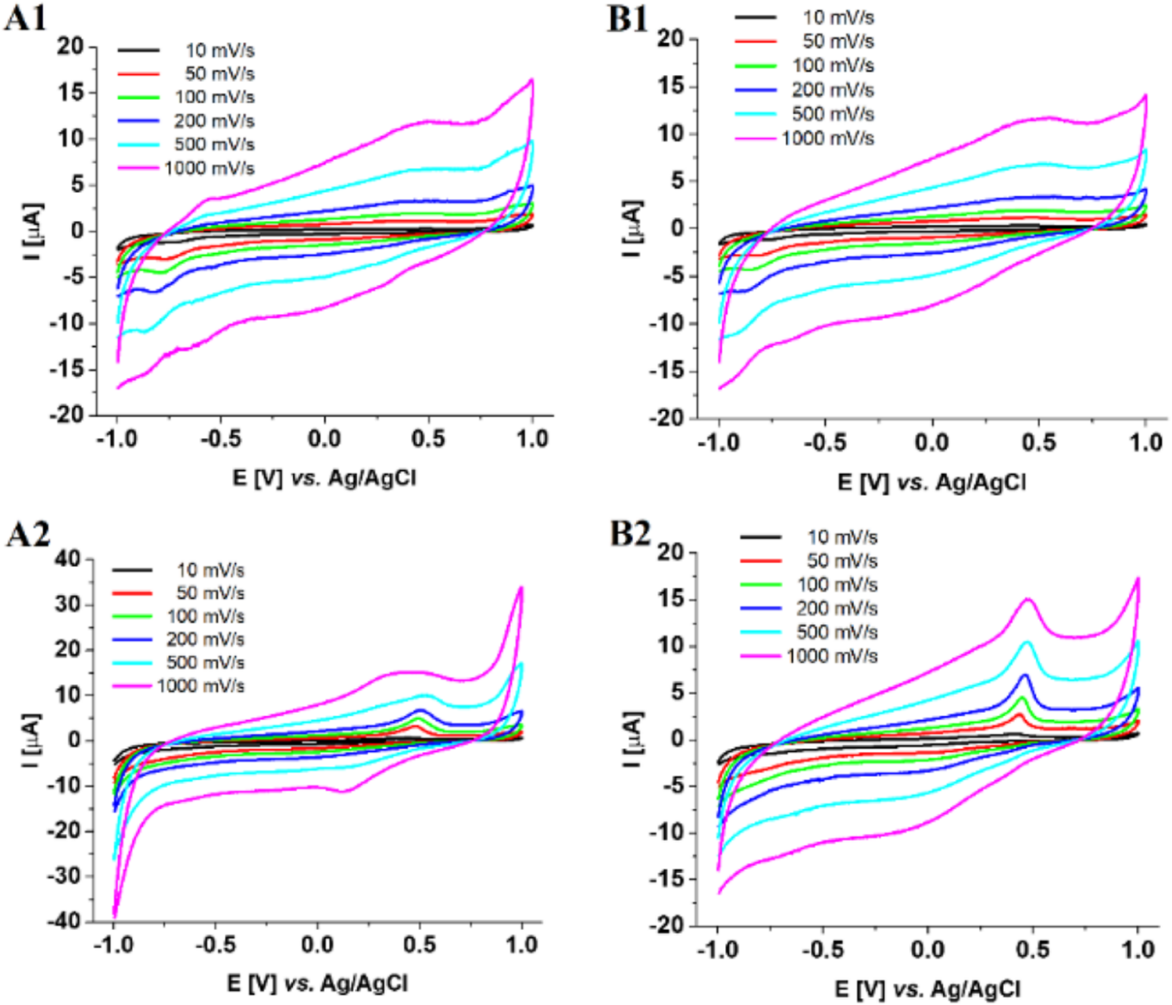
Cyclic voltammetric profiles of (A1) 1 mM **5L**; (A2) 1 mM **Cu5L**; (B1) 1 mM **6L**; and (B2) 1 mM **Cu6L**. CV of the compounds was performed with NaNO_3_ (0.1 M) as a supporting electrolyte in aqueous solution at pH = 7.4, and *T* = 298 K.

**Table 2:** Voltammetric data for Cu­(II) complexes at pH 7.4

system	*E*_pa_ Cu(I)/Cu(II) [V]	*E*_pc_ Cu(II)/Cu(I) [V]	Δ*E* [V]	*E*_1/2_ [V]	*E*^0^ [V] (vs NHE)	*E*_pa_ Cu(II)/Cu(III) [V]	*E*_pc_ Cu(III)/Cu(II) [V]
Cu5L	0.354	0.075	0.279	0.214	0.424	0.496	-
Cu6L	0.227	–0.058	0.285	0.084	0.294	0.450	-

The potential values are given versus the Ag/AgCl electrode. The parameters are given for a scan rate of 100 mV/s.

However, as has been proven, the anodic potentials are always less positive for larger peptides than for tripeptides.^45^ The lack of a cathodic peak for the Cu­(III)/Cu­(II) reaction is probably due to the redox-active tyrosine residue, which is responsible for the quenching of this process. This phenomenon has been proven in the literature for Cu­(II)-Aβ(4–16) complexes.^41,42^ In addition, both complexes show an additional cathodic peak at negative potentials (Table 2). This peak can be attributed to the Cu­(II)/Cu­(I) reduction reaction.^43^ The calculated Δ*E* values for this process as well as the shape of the obtained voltammograms suggest that both complexes are characterized by one quasireversible process (Table 2 and Figure 4A2,B2).

To confirm the potential reduction of the Cu­(II) ion in the complex after the addition of ascorbic acid, we performed EPR measurements. Different concentrations of Asc (0.25, 0.5, 1.0, and 5.0 mM) were used to determine how the concentration of this compound affects the level of reduction of the Cu­(II) ion. If the Cu­(II) ion is reduced to Cu­(I), then the EPR signal should not be observed. No significant changes in the spectra were observed after the addition of 0.25 and 0.5 mM Asc to the complex. However, the addition of 1 mM Asc contributed to the potential reduction of Cu­(II) EPR signal by one-fourth (Table 3). Increasing the Asc concentration to 5 mM caused a 75% reduction in the metal ion. This means that the Cu­(II) ion in the tested complex is susceptible to reduction only under certain conditions.

**Table 3:** Estimated Percentage of Reduced Cu­(II) Ion in the Complex after Addition of Ascorbic Acid (Asc) to the System Is Based on the Recorded EPR Spectra

studied system	*C*_Asc_ [mM]	reduced Cu(II) ions [%]
Cu(II)-Ac-KGHGNGEEGTPTVHNEYH-NH_2_ (Cu6L)	0.25	∼5
	0.50	∼5
	1.00	∼25
	5.00	∼75

The electron transfer from a reducing agent (in our case ascorbate) to molecular oxygen catalyzed by a redox-active metal ion (Cu­(II) ion in **Cu6L** system) results in the formation of reactive oxygen species, in particular the hydroxyl radical (Scheme 1).^46^ We have demonstrated the feasibility of the aforementioned reaction below and identified its primary product.

**Scheme 1:**
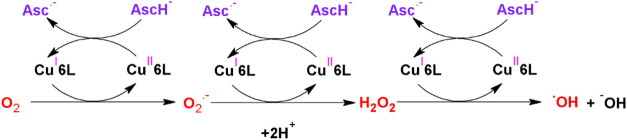
Mechanism of Hydroxyl Radical Generation Catalyzed by Cu Ions from the **Cu6L** System^a^

a The **Cu6L** system reacts with ascorbic acid, then the Cu­(II) ion is reduced to Cu­(I), and the ascorbate anion is converted to ascorbyl radical. Next, the Cu­(I) ion reduces dioxygen in a one-electron reaction to the superoxide anion radical, while itself being oxidized to Cu­(II). In subsequent redox cycles, hydrogen peroxide and hydroxyl radical are formed, respectively.

### Reactive Oxygen Species Production

The hydroxyl radical (^•^OH) is one of the most dangerous reactive oxygen species (ROS) due to its strong oxidizing properties resulting from its high oxidation potential *E*°(^•^OH/H_2_O) = 2.8 V. This value of the redox potential is only slightly lower than that of fluorine.^47^ This makes the reactions involving hydroxyl radicals very fast, with nearly zero activation energy. Consequently, hydroxyl radicals are not selective and can react with almost all organic biomolecules.^48^

To test the activity of **Cu6L** in ^•^OH generation, we performed three experiments (ascorbate consumption, 2-hydroxyterephthalic acid production, and spin trapping). Reactions were carried out in the presence of various molecules that may be present in the large intestine such as hydrogen peroxide (H_2_O_2_) and ascorbic acid (Asc).

During the reaction with Asc at pH 6.8, the generation of hydroxyl radical was monitored by the decrease of the maximum absorption band for Asc at 265 nm (ε_Asc_ = 145,000 M^–1^cm^–1^).^49^ The addition of H_2_O_2_ to Asc drives the reaction toward ROS production, which has been confirmed in the literature.^50^ The reaction of **Cu6L** with H_2_O_2_ is completed after 1 min, proving the high ability of the investigated system to produce free radicals. Cu­(II) complexes and free metal ions without H_2_O_2_ also participate quite effectively in the oxidation of Asc and the formation of ^•^OH, which can be presented in the following order: **Cu6L** (3:1) > **Cu6L** (2:1) > Cu­(II) > **Cu6L** (1:1) (Figure 5A). The slowest reaction recorded for the **Cu6L** system in 1:1 M:L molar ratio was completed after 6 min.

**Figure 5:**
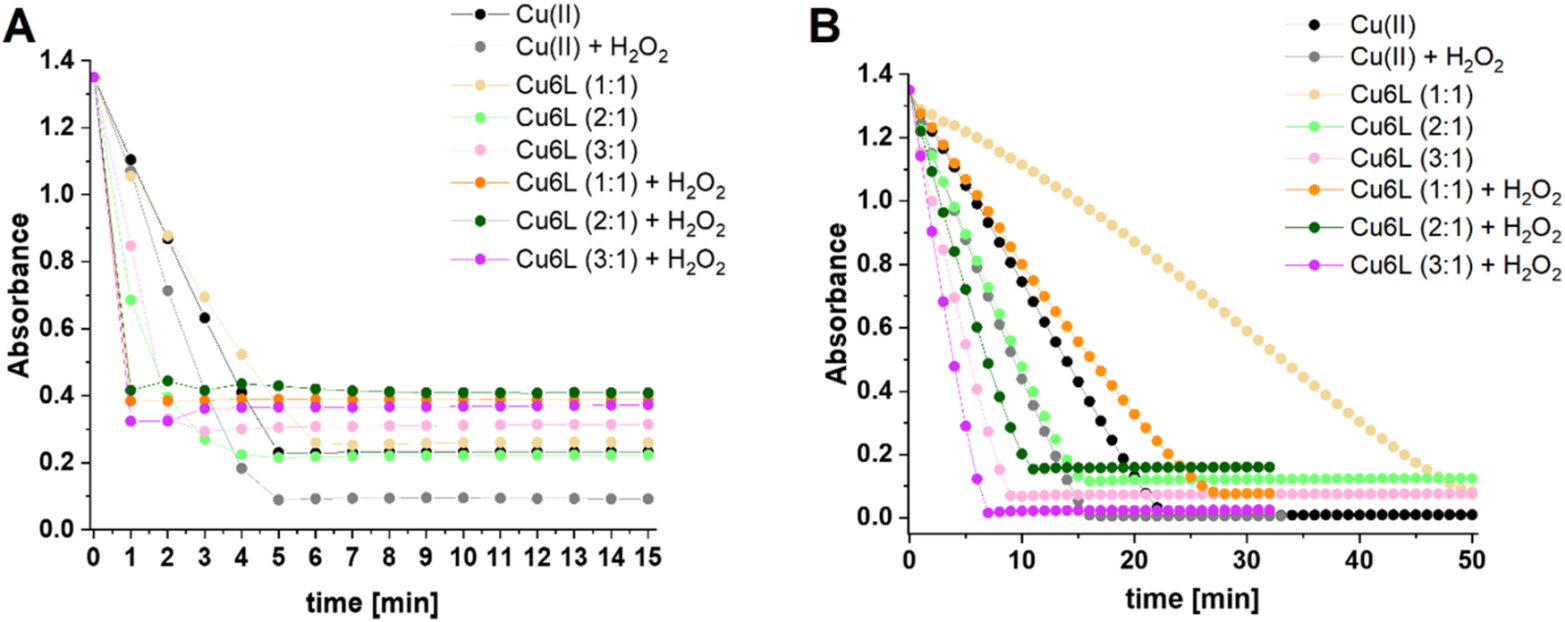
Ascorbic acid consumption test with addition of **Cu6L** system (1:1, 2:1, and 3:1 M:L molar ratios) or uncomplexed Cu­(II) ions, where (A) [Cu­(II)] = 10 μM; [L] = 12 μM and (B) [Cu­(II)] = 5 μM; [L] = 6 μM; with/without H_2_O_2_ at pH 6.8 as a function of time recorded by UV–vis spectroscopy.

The same experiment performed at half the concentration of Cu­(II) ions and ligand slowed down the reaction of ascorbic acid consumption. We can see that decreasing concentration causes the reaction **Cu6L** (3:1) + H_2_O_2_ to complete after 7 min (Figure 5B). This is the fastest reaction among all of those performed. The rate of ascorbate consumption is lower when the M:L molar ratio decreases. The reactions of complexes with hydrogen peroxide are faster than those without the addition of a reducing agent. Again, the slowest reaction is that of **Cu6L** (1:1), which is completed only after 50 min of measurement. Thus, reducing the concentration of the complex has a significant effect on the rate of ascorbate oxidation and ROS production.

As the next step, we selected terephthalic acid as a specific probe for detecting hydroxyl radicals by luminescence.^51^ Figure 6A–C shows the calculated differences in fluorescence intensity (at 425 nm) between one minute and one hour of measurement for the **6L** ligand and its **Cu6L** system (in the 1:1, 2:1, and 3:1 metal-to-ligand molar ratios) in the presence of H_2_O_2_, Asc, or H_2_O_2_/Asc mixture. ^•^OH formation is observed for the **6L** ligand and its **Cu6L** system in the presence of each studied molecule and their mixture. However, similarly to other Cu­(II) complexes with FomA protein fragments,^25,27^ the **Cu6L** system in the presence of H_2_O_2_, Asc, or H_2_O_2_/Asc generates a much higher level of free radicals than the ligand. The results obtained for the **Cu6L** system with the addition of H_2_O_2_ or Asc indicate that the higher the M:L molar ratio, the greater the generation of hydroxyl radicals.

**Figure 6:**
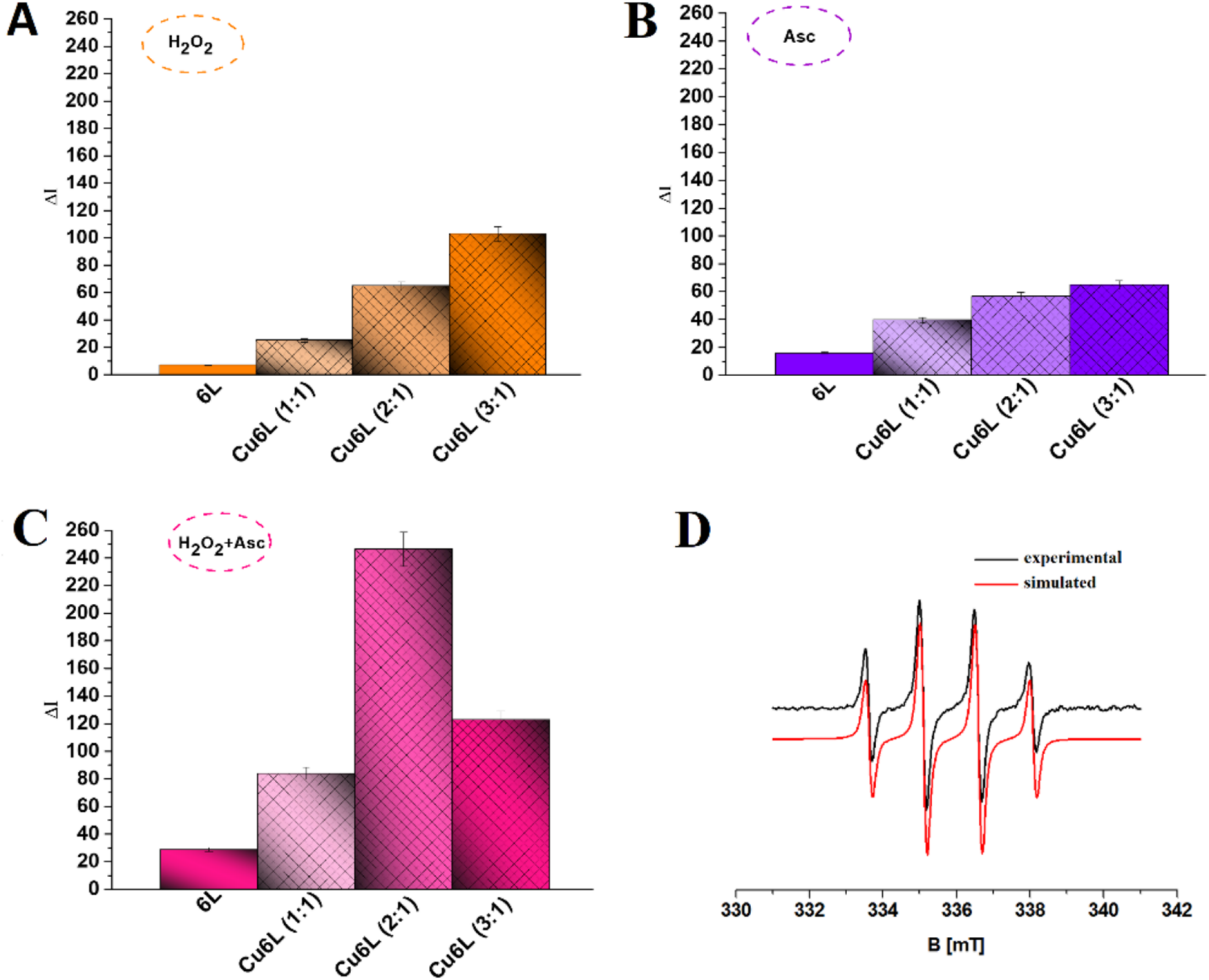
Generation of hydroxyl radical by **Cu6L** system demonstrated in two experiments: 2-hydroxyterephthalic acid production recorded by luminescence and expressed as a comparison of the difference in fluorescence intensity (Δ*I* = *I*_*t*=1h_ – I_*t*=1min_) (at 425 nm) for the ligand and its Cu­(II) complex (in 1:1, 2:1, and 3:1 M:L molar ratios) in the presence of (A) H_2_O_2_, (B) Asc, or (C) H_2_O_2_/Asc mixture and (D) EPR spin trapping which results in an EPR spectrum of the DMPO spin adduct formed during the reaction of the **Cu6L** system (1:1 M:L molar ratio, [L] = 50 μM) in the presence of H_2_O_2_ (C = 25 μM).

The exception is the reaction carried out in the presence of H_2_O_2_/Asc mixture, where the **Cu6L** system in a 2:1 M:L molar ratio leads to the formation of the highest concentration of ^•^OH. Comparing Cu­(II) complexes with different fragments of FomA protein, it can be seen that the **Cu6L** system (in the 1:1 and 2:1 M:L molar ratio) in the presence of H_2_O_2_ or Asc is as efficient in producing ^•^OH as the Cu­(II)-Ac-PTVHNEYH-NH_2_ (**Cu5L**) complex.^25^ The calculated pCu values (−log­[Cu­(II)_free_]) for the **Cu6L** (pCu = 6.63 at pH 6.8, pCu = 8.16 at pH 7.4) and **Cu5L** (pCu = 6.28 at pH 6.8, pCu = 8.40 at pH 7.4) systems are similar, indicating similar stability of both complexes. The lack of a significant difference in the concentration of unbound Cu­(II) ions suggests that both complexes should generate similar levels of ROS, which is consistent with our results. Analogous behavior of the complex is observed in a 2:1 M:L molar ratio with H_2_O_2_/Asc mixture. In turn, the Cu­(II)-cyclo­(KGHGNGEEGTPTVHNE) (**Cu4L**) complex (in a 1:1 M:L molar ratio and in the presence of H_2_O_2_) mimicking loop no. 4 of the FomA protein seems to be almost 6 times more efficient in producing hydroxyl radicals than the **Cu6L** and **Cu5L** systems.^27^

Adding H_2_O_2_/Asc mixture to the complexes causes the **Cu6L** system (1:1 M:L molar ratio) to show lower activity (about 35%) in ^•^OH production than the **Cu5L** complex. Because the **Cu6L** system shows a very similar activity in ROS production to the Cu­(II)-Ac-PTVHNEYH-NH_2_ (**Cu5L**) complex (**5L** fragment contained in the **6L** peptide sequence), and that the **6L** peptide is fragmented into the EGTPTVHNEYH after incubation with Asc, it can be concluded that the **5L** fragment is mainly responsible for ROS production. In turn, the generation of huge amounts of ^•^OH by the **Cu4L** complex may result from the conformational restriction resulting from the peptide cyclization, which most likely favors the Cu­(II)/Cu­(I) redox conversion.^52^

The calculated first-order rate constants for the **6L** ligand and its Cu­(II) complex (**Cu6L**) in all tested (1:1, 2:1, and 3:1) M:L molar ratios in the presence of H_2_O_2_, Asc, or H_2_O_2_/Asc are presented in Table 4. The obtained values indicate that Cu­(II) complexes in a 3:1 M:L molar ratio in the presence of H_2_O_2_ or Asc generate the ^•^OH radical the fastest. In the case of H_2_O_2_/Asc mixture, the Cu­(II) complex in a 2:1 M:L molar ratio is the most effective in producing hydroxyl radicals (1.76(4) × 10^–1^ s^–1^). Comparing the **Cu6L** system with other complexes containing FomA protein fragments, it can be concluded that the production of hydroxyl radical by **Cu6L** is characterized by the slowest first-order rate constant (except for the reaction in a 2:1 M:L molar ratio and in the presence of a H_2_O_2_/Asc mixture). Under these conditions, the **Cu6L** system generates ^•^OH faster than the Cu­(II)-Ac-KGHGNGEEGTPTVHNE-NH_2_ (**Cu3L**) complex and its cyclic counterpart (**Cu4L)** (*k*_obs_ = 8.2(1) × 10^–2^ and 8.4(7) × 10^–2^, respectively).^27^ The Cu­(II)-Ac-PTVHNEYH-NH_2_ (**Cu5L**) complex appears to produce radicals at the highest rate in a 2:1 M:L molar ratio in the presence of H_2_O_2_, Asc, and their mixture, and in a 1:1 M:L molar ratio with Asc.^25^ In other cases, the complex with the cyclopeptide (**4L**) appears to be more efficient.

**Table 4:** First-Order Rate Constants *k*_obs_ [s^–1^] for the Formation of TAOH with Added Ligand and Its Cu­(II) Complex in the Presence of H_2_O_2_, Asc, or H_2_O_2_/Asc for the First 5 min of the Reaction

		H_2_O_2_	Asc	H_2_O_2_/Asc	literature
detector	system	*k*_obs_ [s^–1^]			
TA	3L	1,6(6) × 10^–2^	1,7(7) × 10^–2^	1,2(7) × 10^–2^	^27^
	Cu3L(1:1)	1,8(7) × 10^–2^	2,5(6) × 10^–2^	1,8(5) × 10^–1^	^27^
	Cu3L(2:1)	2,7(5) × 10^–2^	3,7(8) × 10^–2^	8,2(1) × 10^–2^	^27^
	4L	1,5(4) × 10^–2^	1,6(4) × 10^–2^	1,3(5) × 10^–2^	^27^
	Cu4L(1:1)	2,6(8) × 10^–2^	3,1(5) × 10^–2^	1,8(6) × 10^–1^	^27^
	Cu4L(2:1)	6,3(6) × 10^–2^	3,8(7) × 10^–2^	8,4(7) × 10^–2^	^27^
	5L	6,8(1) × 10^–3^	5,09(2) × 10^–3^	1,71(6) × 10^–2^	^25^
	Cu5L(1:1)	2,5(1) × 10^–2^	5,1(2) × 10^–2^	1,02(3) × 10^–1^	^25^
	Cu5L(2:1)	7,8(2) × 10^–2^	9,4(2) × 10^–2^	2,64(8) × 10^–1^	^25^
	6L	3,6(1) × 10^–3^ ± 2.06 × 10^–5^	4,0(5) × 10^–3^ ± 1.26 × 10^–5^	5,4(1) × 10^–3^ ± 2.03 × 10^–5^	this article
	Cu6L(1:1)	1,22(1) × 10^–2^ ± 1.50 × 10^–4^	2,6(1) × 10^–2^ ± 5.91 × 10^–4^	3,15(4) × 10^–2^ ± 1.47 × 10^–4^	this article
	Cu6L(2:1)	2,05(1) × 10^–2^ ± 1.02 × 10^–4^	3,20(4) × 10^–2^ ± 7.56 × 10^–4^	1,76(4) × 10^–1^ ± 2.83 × 10^–3^	this article
	Cu6L(3:1)	1,01(2) × 10^–1^ ± 2.57 × 10^–3^	1,78(6) × 10^–1^ ± 3.22 × 10^–3^	5,2(1) × 10^–2^ ± 3.03 × 10^–4^	this article

Direct identification of the ROS generated during the reaction of the tested Cu­(II)-Ac-KGHGNGEEGTPTVHNEYH-NH_2_ (**Cu6L**) system with H_2_O_2_ was provided with EPR measurements of the DMPO spin trapping. The characteristic spectrum of the DMPO-^•^OH spin adduct (Figure 6D), with the hyperfine splitting parameters obtained from computer simulation, is typical for trapped hydroxyl radicals (*A*_izo_(N) = 1.49 mT and *A*_izo_(H) = 1.48 mT).^53^

In turn, the use of gel electrophoresis allowed us not only to confirm the presence of the ^•^OH as in the above experiments but also to identify other formed ROS. Therefore, specific ROS scavengers such as DMSO, sodium azide, and potassium iodide were used to detect ^•^OH, ^1^O_2_, and O_2_^•‑^, respectively. Additionally, it was possible to observe DNA damage induced by oxidative stress. The **Cu6L** system in the presence of H_2_O_2_ leads to single-strand breaks in DNA (about 70% of form II) (Figure 7A1,B1, and lane 3). This complex seems to be more aggressive toward DNA compared to the Cu­(II)-Ac-KGHGNG-NH_2_ system (**Cu1L**) (about 35% of the circular form of DNA) (Figure S13).^23^ However, Cu6L is less active in DNA damage than the Cu­(II)-Ac-PTVHNE-NH_2_ complex (**Cu2L**), which leads to double-strand breaks in DNA (about 40% of form III) (Figure S13). Other FomA protein fragments (Ac-KGHGNGEEGTPTVHNE-NH_2_ (3L), cyclo­(KGHGNGEEGTPTVHNE) (**4L**), Ac-PTVHNEYH-NH_2_ (**5L**)) coordinated with Cu­(II) ions were more active in generating ROS and damaging DNA (since linear form of DNA was formed in all cases) than **Cu1L** and **Cu6L** systems (Figure S13).^25,27^ In experiments for the **Cu3L**-**Cu5L** complexes, an even lower H_2_O_2_ concentration (25 μM) was used because the DNA damage was too high at a 50 μM H_2_O_2_ concentration. All these complexes produced singlet oxygen and hydroxyl radical, which cause DNA damage mentioned above.

**Figure 7:**
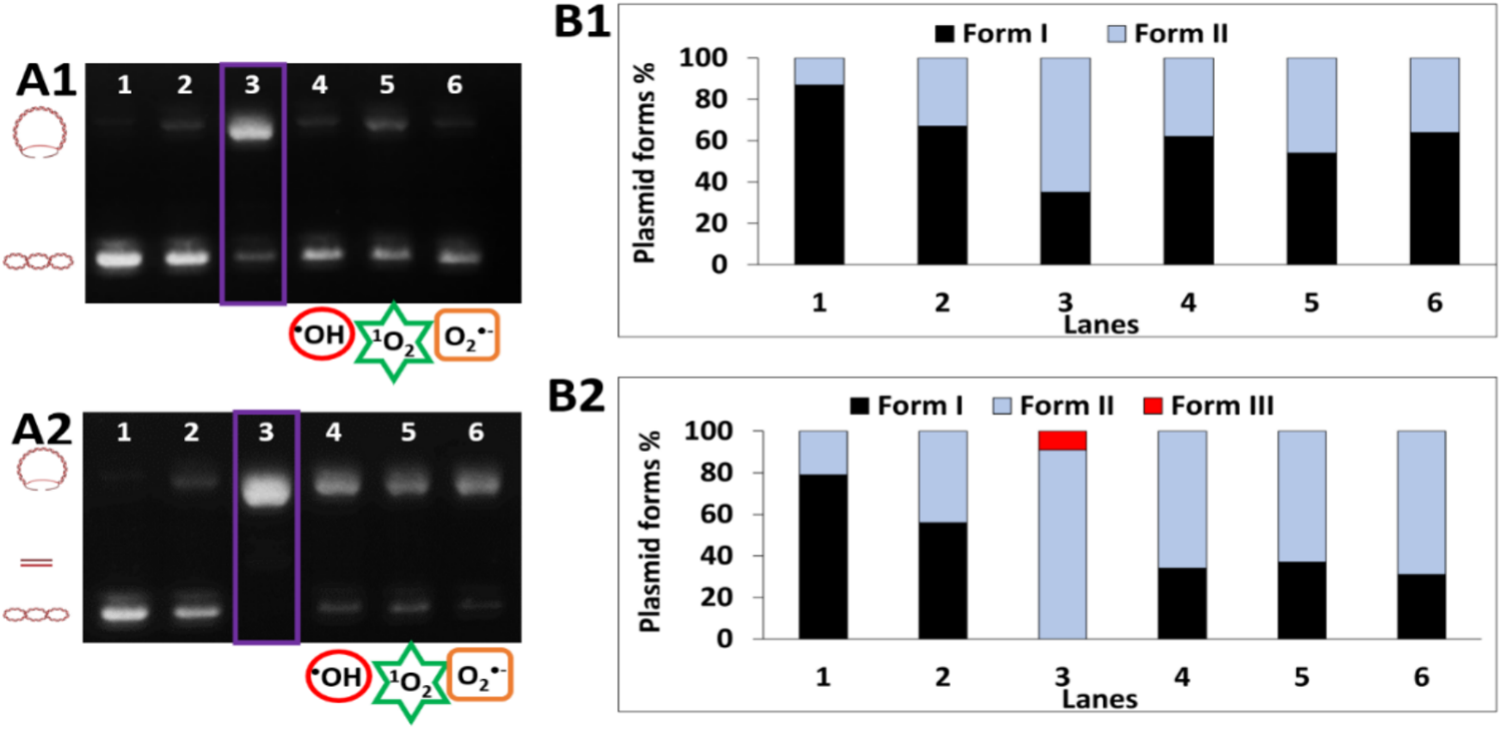
Agarose gel electrophoresis of pBR322 plasmid cleavage by the **Cu6L** system (C = 50 μM) in the presence of 50 μM H_2_O_2_ (A1) and 100 μM Asc (A2) at pH 6.8. Lanes: **1:** plasmid; **2:** plasmid + **Cu6L**; **3:** plasmid + **Cu6L** + H_2_O_2_/Asc; **4:** plasmid + **Cu6L** + H_2_O_2_/Asc +0.14 M DMSO; **5:** plasmid + **Cu6L** + H_2_O_2_/Asc + 40 mM NaN_3_; **6:** plasmid + **Cu6L** + H_2_O_2_/Asc + 8 mM KI; (B) densitometric analysis of the presented electropherograms.

In the case of **Cu6L**, an additional superoxide anion radical was detected. Moreover, the **Cu2L** complex causes the greatest DNA damage among all of the studied complexes with FomA protein fragments (Figure S13). This indicates that in this complex, the Cu­(II) ion is most loosely bound by the ligand donor atoms, which allows for easy cyclization of the Cu­(II)/Cu­(I) ion and the production of the largest amount ROS (hydroxyl radical, singlet oxygen and perhaps other ROS not identified by us), which lead to DNA damage. Surprising results were obtained for the **Cu5L** and **Cu6L** systems. Although these complexes produce ^•^OH at a comparable level, the DNA damage induced during the reaction with the addition of these complexes is different. The **Cu5L** complex is more aggressive toward DNA, which can most likely be explained by the production of larger amounts of different, various types of ROS (possibly also those not detected by us in the reactions) than in the case of the **Cu6L** system. The addition of Asc to the **Cu6L** system leads not only to the formation of a circular form of DNA but also to double-strand breaks in DNA (Figure 7A2, B2, and lane 3). Moreover, the superhelical form of DNA is not present in the sample (Figure 7A2, B2, and lane 3).

### Cellular ROS Production and Consequences for Host Cells

In this paper, we check the influence of FomA protein fragments (**5L**, **6L**) and their Cu­(II) complexes on cellular reactive oxygen species production. The data has been compared with the literature. At first, we evaluated the cytotoxic effect of Cu­(II) ions and **5L**, **6L**, **Cu5L**, and **Cu6L** toward mouse colon carcinoma (CT26—a model cell line of the human colon carcinoma^23,54^ ) used to compare with the cytotoxicity of **1L**, **2L**, **Cu1L**, **Cu2L** ^23^ and **3L**, **4L**, **Cu3L**, **Cu4L** ^27^ compounds. The IC_50_ values were calculated (Table 5) to choose a nontoxic concentration of the studied peptide fragments and their Cu­(II) complexes, allowing research on ROS generation. Interestingly, Cu­(II) ions showed the highest cytotoxicity among the described substances (Table 5). In the next step of our study, we decided to verify if Cu­(II) complexes with FomA protein fragments can be accumulated inside the mouse colon carcinoma cells (CT26) or rather they are attached to the cells’ surface.

**Table 5:** IC_50_ Values (mM) and Copper Uptake [ng Cu/mg Protein ± SD] for CT26 Cell Lines after 24 h of Treatment with Ligands and the Copper­(II) Complexes with Peptide Motifs and Cu­(II) Ions

compound	IC_50_ [mM] ± SD	ng Cu/mg protein ± SD	literature
**1L**	1.03 ± 0.12		^23^
**2L**	1.19 ± 0.02		^23^
**3L**	1.08 ± 0.15		^27^
**4L**	1.01 ± 0.10		^27^
**5L**	1.57 ± 0.22		
**6L**	1.47 ± 0.34		
**Cu1L**	1.09 ± 0.06	136 ± 22	^23^
**Cu2L**	1.08 ± 0.19	112 ± 31	^23^
**Cu3L**	1.12 ± 0.10	125 ± 14	^27^
**Cu4L**	1.23 ± 0.12	119 ± 18	^27^
**Cu5L**	1.58 ± 0.52	18 ± 5.2	
**Cu6L**	1.73 ± 0.62	19 ± 8.1	this paper
**Cu(II)**	0.50 ± 0.01	1130 ± 80	this paper

The intracellular Cu concentration for the CT26 cell line treated with **Cu5L**, **Cu6L**, and free copper­(II) ions at IC_50_ concentrations was assessed by ICP-MS analysis. The compounds were incubated with the cells for 5 min and 4, 12, and 24 h, and the copper amount was expressed as ng Cu/mg protein (Table 5 and Figure 8).

**Figure 8:**
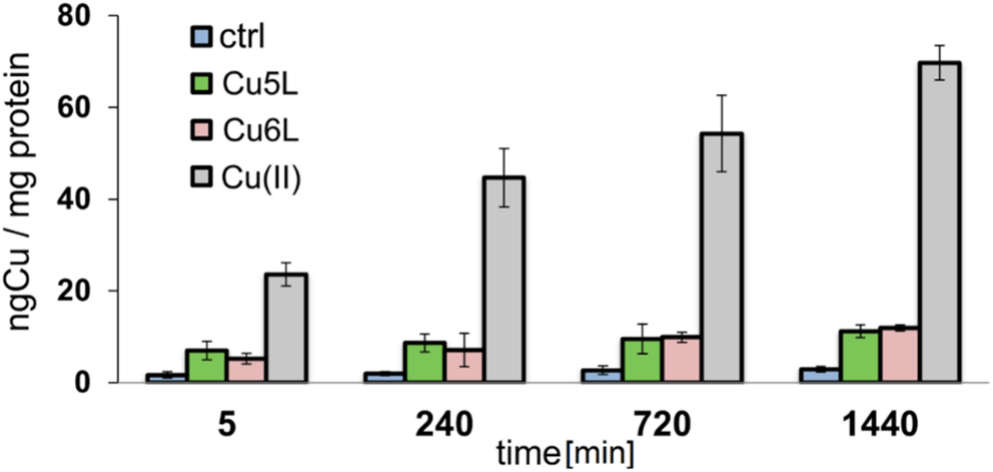
Cellular copper concentration in CT26 cell line using ICP-MS method after 5 min and 4, 12, and 24 h of incubation with **Cu5L**, **Cu6L**, and free copper­(II) at IC_50_ concentration.

The obtained results clearly indicate that the studied copper­(II) complexes did not enter the cells effectively. Extension of incubation time (from 5 min to 24 h) did not result in a significant difference between copper level inside the cells studied here with (**Cu5L**, **Cu6L**) and literature (**Cu1L**, **Cu2L**, **Cu3L**, and **Cu4L**) complexes (Figure 8).

At the same time, the accumulation of free copper­(II) cations inside the cells increased five times compared to the control and copper­(II) complexes. Uncoordinated copper­(II) cations entered the cells much easier than copper complexes with FomA fragments, which are consistent with their higher cytotoxic activity (Table 5). This phenomenon is not surprising as it is well known that the protein 1 (CTRL-1), which exhibits high affinity for copper uptake, transports Cu cations into the cells at the plasma membrane.^55^

### Time- and Dose-Dependent Cellular ROS Generation

Reactive oxygen species production by CT26 cells was evaluated using H2DCF-DA-2′,7′-dichlorodihydrofluorescein diacetate, nonfluorescent form, that can be oxidized by ROS to a fluorescent DCF-2′,7′-dichlorofluorescein.^56^ The level of ROS generation by the investigated Cu­(II) compounds can be followed by monitoring the changes in fluorescence intensity. We applied H_2_O_2_ as positive control. Since it is well known that ROS production highly depends on the concentration of the active compounds, we have screened several concentrations (*C* = 0.1, 0.01, 0.001 mM), lower than the calculated IC_50_ values. Additionally, various incubation times (5 min and 0.5, 4, 12, 24 h) were applied (Figure 9).

**Figure 9:**
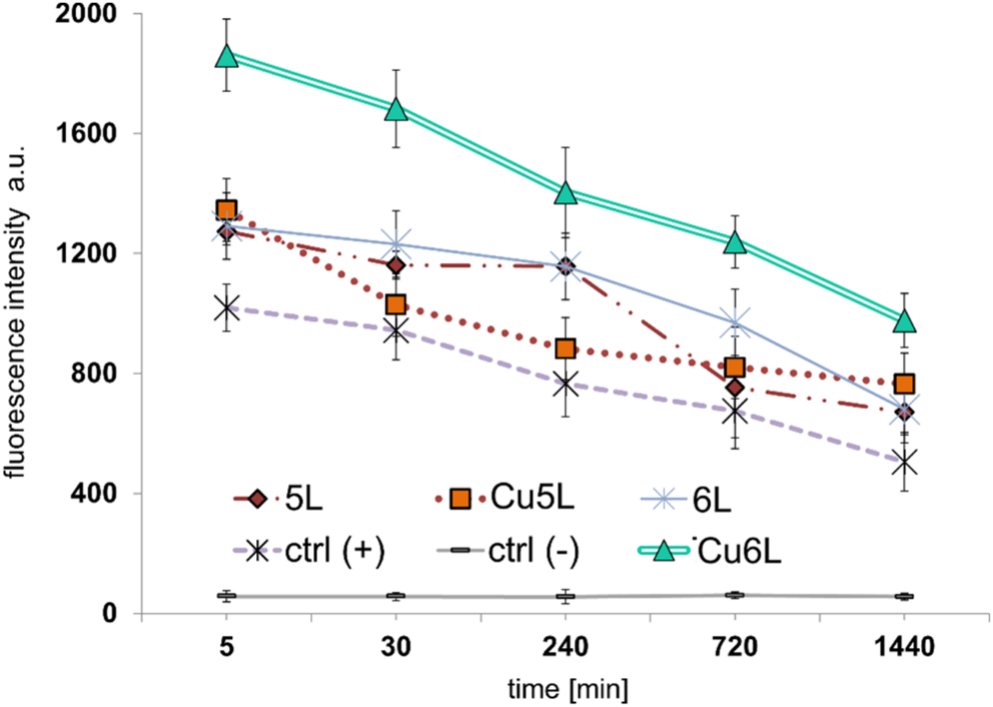
Changes of fluorescence intensity for CT26 cells incubated with **5L**, **Cu5L**, and **6L**, **Cu6L** at various concentrations after 5 min and 0.5, 4, 12, and 24 h of incubation, detection with H2DCF-DA probe, ctrl­(+): H_2_O_2_ as a positive control, and ctrl(−): cells without copper compounds as a negative control.

The highest level of ROS generation by CT26 cells was noticed after 5 min incubation with all Cu­(II) complexes. With elapsing incubation time, the level of ROS production decreased significantly for all complexes (Figure 9). It can be concluded that CT26 cells produce the highest ROS level right after contact with the copper-FomA fragments. Since it is well known that reactive oxygen species are kinetically unstable, a decrease in the ROS level noticed during the elongation of incubation time seems reasonable. We checked that all copper­(II) complexes stimulated cells to higher ROS production than the native FomA fragments (**1L**-**6L**) and free copper­(II) ions for all incubation times. Interestingly, the **Cu6L** system was responsible for the highest level of ROS production than **Cu5L** (Figures 9, 10, and S14–S19) and the ones (**Cu1L-Cu4L**) reported in the literature.^23,27^ Obtained results differ from those presented above (chapter entitled **Reactive Oxygen Species Production**) where we proved that **Cu5L** is the most powerful ROS generator. However, we think that in biological environments, **6L** peptide undergoes fragmentation and different species of Cu­(II)-peptide fragments including **Cu5L** are formed, being responsible for such high ROS generation.

**Figure 10:**
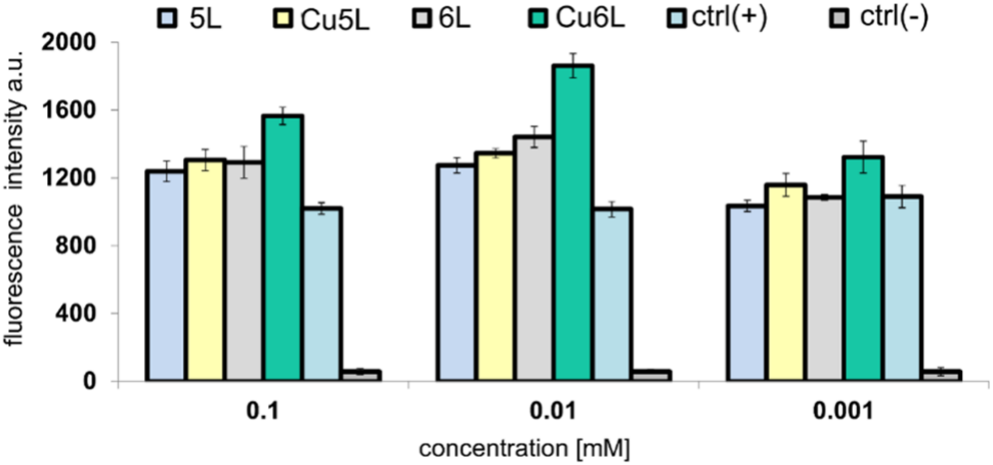
Reduction of fluorescence intensity of CT26 cells incubated with **Cu5L** and **Cu6L** at different concentrations (0.1, 0.01, and 0.001 mM) observed using H2DCF-DA probe.

As can be seen in Figures 10 and S14–S19, the observed influence of the Cu compounds on ROS production is strongly nonlinear and passes through a maximum for the value of 0.01 mM for all studied here and literature^23,27^ compounds. Once the concentration exceeds 0.01 mM, the reactive oxygen species’ level decreases. A very similar relationship between the oxidative stress and the level of silver and zinc oxide nanoparticles in the human epithelial colorectal adenocarcinoma cells (Caco-2) was explained by Song and co-workers.^57^

Reactive oxygen species can be produced in two different signaling pathways: intracellular and extracellular.^58^ In our case, an extracellular pathway to ROS production seems to be the most probable because of the low penetration of the cell by the studied complexes. They probably bind to the specific receptors covering the cells’ surface. Consequently, various types of intracellular signals can be generated owing to these receptor–ligand interactions,^59,60^ which may lead to high levels of intracellular ROS generation.^58^

### Lipid Peroxidation

The radicals overgenerated in the cells have damaging consequences for a human organism, leading to lipid peroxidation,^61^ while reactive aldehydes such as 4-hydroxynonenal (4-HNE) and malondialdehyde (MDA) are produced.^62,63^ Notably, the increase in intracellular MDA concentration is labeled with tissue damage.^64^ MDA reacts with nucleic acids and proteins, leading to changes in their structure and properties, especially to mutagenesis processes and, eventually, carcinogenesis.^65,66^ It has also been established that lipid peroxidation impacts colorectal cancer development based on increased level of MDA concentration in colorectal tissues.^67^

The concentration of MDA, a lipid peroxidation marker, inside the CT26 cells was monitored after 5 min and 0.5, 4, 12, 24, and 48 h treatment with **5L**, **6L**, **Cu5L**, **Cu6L**, H_2_O_2_ (Figure 11) and compared with literature data (**Cu1L**, **Cu2L**, **Cu3L**, and **Cu4L**).^23,27^ Hydrogen peroxide was used as a positive control. The obtained results show a gradual increase of MDA concentration for all studied (**Cu5L**, **Cu6L**) (Figure 11) and literature compounds (**Cu1L**-**Cu4L** ^23,27^ ). ROS produced by carcinoma cells after treatment with the **Cu6L** system led to the formation of the highest amount of MDA, regardless of the incubation time and concentration of all studied and literature compounds. The correlation between produced ROS and the concentration of MDA was additionally confirmed with the experiments involving N-acetylcysteine (NAC, 5 mM). The MDA concentration was determined in cells, which were preincubated with NAC, and further treated for 24 h with **5L**, **6L**, **Cu5L**, **Cu6L**, Cu­(II), and H_2_O_2_ at the most effective concentration of 0.01 mM. The results are shown in Figure 11. It shows that the production of ROS is inhibited by NAC for all of the studied compounds. The strongest influence of NAC was observed for **Cu5L** and **Cu6L** compared with the literature results for compounds **Cu1L**-**Cu4L**.^23,27^ An almost 2-fold decline in MDA concentration was observed for these compounds.

**Figure 11:**
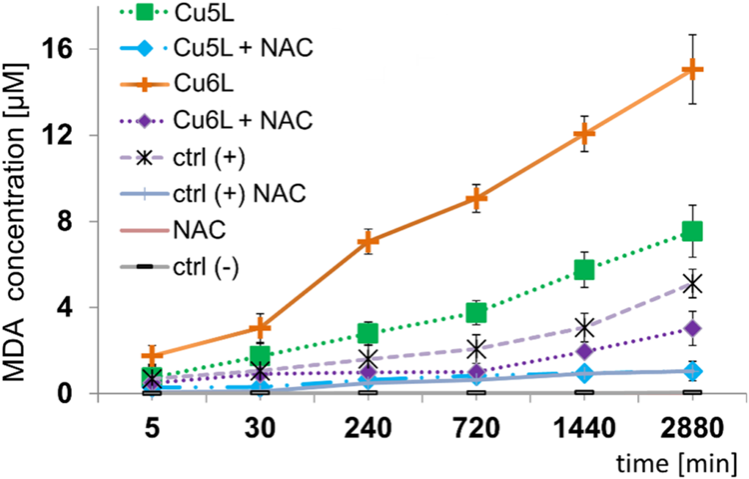
Changes of MDA concentration in CT26 cells incubated with **Cu5L** and **Cu6L** (0.01 mM) in the presence and absence of NAC (5 mM). The results were obtained after 5 min and 0.5, 4, 12, 24, and 48 h of incubation. Ctrl (+) H_2_O_2_ (0.01M).

The highest increase of MDA concentration in cells was observed for the compounds at a concentration of 0.01 mM (Figure 12), which remains in agreement with the peak production of ROS induced from the fluorescence measurements.

**Figure 12:**
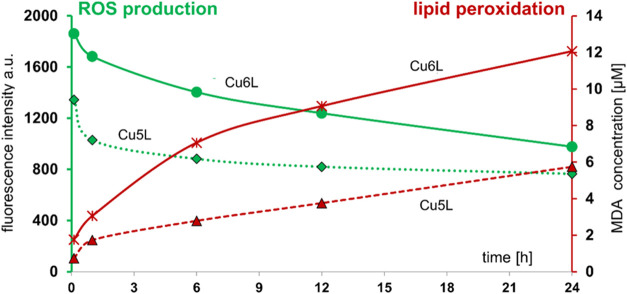
Changes of MDA concentration in CT26 cells after treatment with **5L**, **6L**, **Cu5L**, **Cu6L**, Cu­(II), and H_2_O_2_ (C = 0.01 mM) compounds after 5 min and 0.5, 6, 12, and 24 h of incubation. The green part of the plot represents ROS production, while the red part represents lipid peroxidation.

Indeed, this observation is directly connected with a high concentration of ROS, which leads to the development of lipid-based radicals and, consequently, to the acceleration of MDA formation. After 24 h of incubation, the ROS level decreased, and a simultaneous increase of MDA concentration was observed. Thus, the lipid oxidation results are consistent with the data regarding the production of the free radicals (*vide supra*).

### Possible Mechanism of Cancerogenic Progression

It is widely recognized that adheres to colorectal cells through adhesin, promoting carcinogenesis.^68–70^ We hypothesize that the coordination of copper­(II) ions (whose concentration increases during inflammation) to bacterial FomA protein changes its structure and triggers cell signaling that leads to significant ROS production (Figure 13).

**Figure 13:**
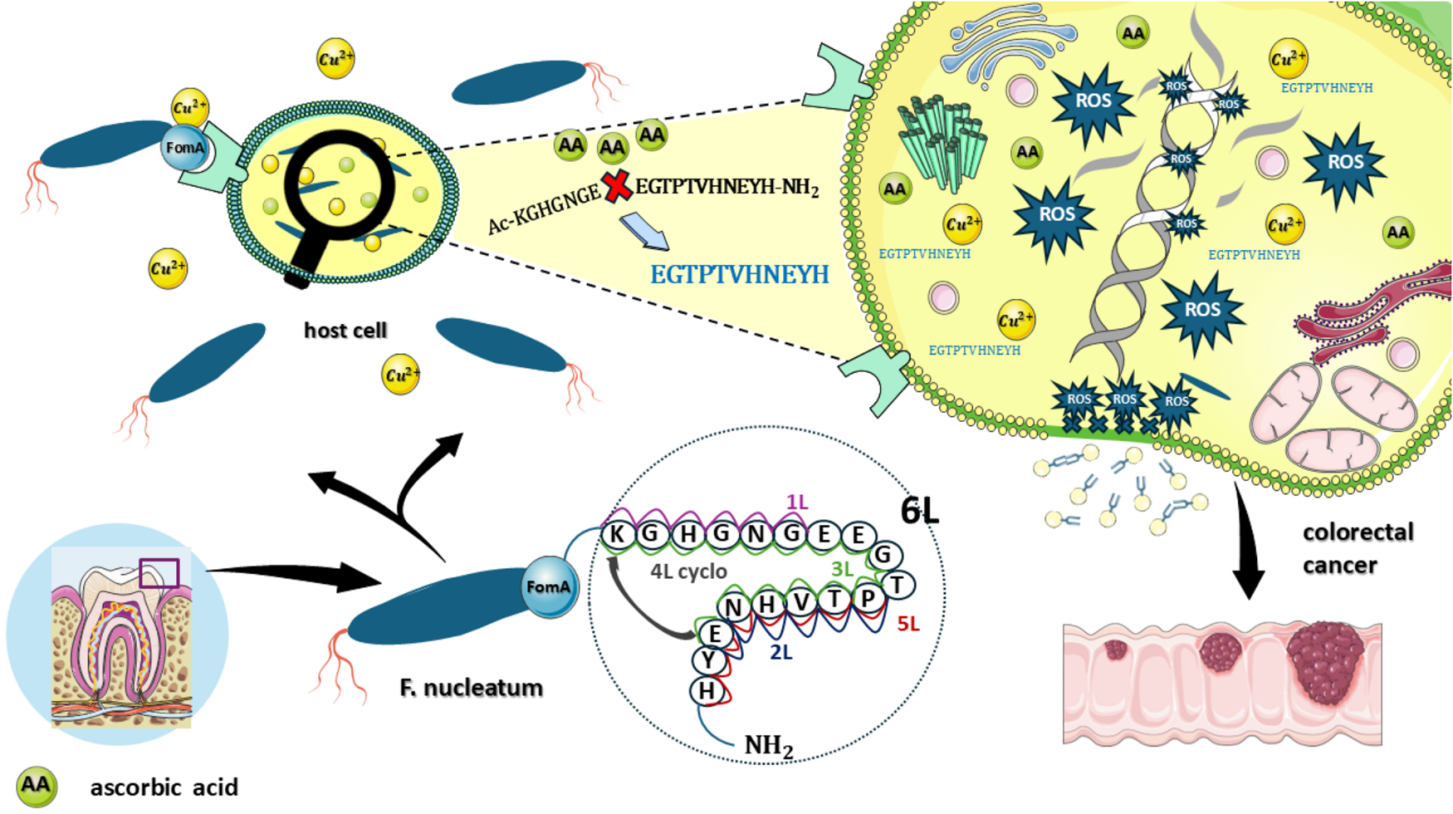
Mechanistic insights into colorectal cancer development driven by ROS generated by the Cu­(II) complex with FomA protein fragments.

Moreover, we proved that FomA protein can be fragmented under specific conditions like the presence of reductants or oxidants, leading to the formation of redox-active Cu complexes, which can be partially accumulated inside the host cell or at least can help transport copper ions inside the cell. Consequently, the generation of ROS results in oxidative stress, causing substantial damage to cellular components, such as lipids or DNA. The oxidation of these lipids increases the concentration of toxic MDA within the cells. Lipid peroxidation and DNA damage may represent one of the stages of the carcinogenesis process. However, a more detailed understanding of the role of ROS and their generation mechanisms within cells is necessary.

## Conclusions

This article describes the results of studies on Ac-KGHGNGEEGTPTVHNEYH-NH_2_—a model peptide derived from the FomA protein. When exposed to the external environment, this **6L** fragment can bind Cu­(II) ions during inflammation and produce ROS, which may be harmful to colon cells and therefore cause the development of colorectal cancer.

The addition of ascorbic acid to the studied complex induces the fragmentation of the **6L** peptide into, among others EGTPTVHNEYH. Remarkably, it retains the ability to bind Cu­(II) ions and generate reactive oxygen species (ROS), which we identified as ^•^OH, ^1^O_2_, and O_2_^•‑^. Furthermore, we observed that the ROS-generating ability of the **Cu6L** system is comparable to the most redox-active **Cu5L** complex (with the Ac-PTVHNEYH-NH_2_ peptide). This peptide sequence is embedded within the **6L** ligand and was the subject of our previous studies. These findings suggest that short peptide fragments may play a crucial, tangible, and significant role in ROS generation within colon cells, which was demonstrated by us in this paper. We confirmed that ROS production leads to oxidative stress, contributing to the increased concentration of toxic MDA in the cells. Consequently, lipid peroxidation by ROS during copper-induced oxidative stress is the primary process underlying colorectal carcinogenesis.

## Experimental Section

### Peptide

Ac-KGHGNGEEGTPTVHNEYH-NH_2_ peptide was purchased from KareBay Biochem. The purity (>95%) was verified by HPLC analysis.

## Coordination Studies

### Potentiometric Measurements

The stability constants for **6L** peptide and its Cu­(II) complexes (**Cu6L**) were calculated from pH-metric titrations performed on a 905 TitrandoMethrom with an InLab Semi-Micro pH electrode from Mettler Toledo. The pH readings were converted into hydrogen ion concentration as described earlier.^71^ The experiments were carried out at 298 K under an argon atmosphere using a total sample volume of 2 mL. As the titrant, CO_2_-free, 0.1 M NaOH was used. The Cu­(II) stock solution was prepared from Cu­(NO_3_)_2_ × 3 H_2_O. The ligand concentration was 0.0015 M, and the metal-to-ligand molar ratios for the studied complex were 1:1, 2:1, and 3:1 (the concentration of the stock solution of Cu­(II) ions was 0.05 M). All measurements were carried out at a constant ionic strength of 0.1 M KNO_3_. Samples were prepared in distilled water (Simplicity UV, Millipore, 18.2 MΩ resistance) in the presence of HNO_3_ to obtain an acidic pH. The samples were titrated twice in the pH range 2.5–10.5. The final results were calculated with SUPERQUAD^72^ and HYPERQUAD^73^ software. The results of the ligand’s titration in the presence of various equivalents of Cu­(II) were analyzed in batch calculations, in which all titration curves were fitted at the same time with one model. The standard deviation values were referred to random errors only. The purity and the exact concentration of the ligand solutions were determined by the Gran method.^74^ The equilibrium reactions of the ligands with protons and Cu­(II) ions are given in the eq 1

1 $$\mathrm{pCu}+\mathrm{qH}+\mathrm{rL}={\mathrm{Cu}}_{p}H_{q}L_{r}$$

where L stands for the studied peptide. The stability constant, β_pqr_, is defined by eq 2

2 $$\beta_{\mathrm{pqr}}=[{\mathrm{Cu}}_{p}H_{q}L_{r}]/{\left[\mathrm{Cu}\right]}^{p}{\left[H\right]}^{q}{\left[L\right]}^{r}$$

#### Safety Statement

Sodium hydroxide (NaOH) and nitric acid (HNO_3_) were used for the pH adjustment. Due to their corrosive nature, all handling was conducted with appropriate PPE and in well-ventilated areas to avoid skin or eye contact and inhalation of vapors.

### Spectroscopic Measurements

The concentrations of the studied solutions were similar to those used in potentiometric studies. The metal-to-ligand molar ratios were 1:1, 2:1, and 3:1. The complexes were measured in the pH range of 3.0–10.5 in 1 and 0.1 cm cuvettes. NaOH and HNO_3_ solutions were used to adjust the pH value. The electron absorption (UV–vis) spectra were recorded on a Cary 60 spectrophotometer (Agilent Technologies) over the spectral range of 200–800 nm, while the circular dichroism (CD) spectra were recorded on a J-715 spectropolarimeter (JASCO) in the range of 190–800 nm. The spectra are expressed in terms of Δε = ε_L_ – ε_R_, where ε_L_ and ε_R_ are the molar absorption coefficients for left and right circularly polarized light, respectively. The electron paramagnetic resonance (EPR) spectra of Cu­(II) complexes were measured on a Bruker spectrometer (Bruker ELEXSYS E500 CW-EPR) at an X-band frequency (9.45 GHz). A solution of ethylene glycol in water was added to each sample (3:10, v:v) to obtain a glass-forming solvent upon freezing with liquid nitrogen. The measurements were carried out at 77 K. The experimental spectra were computer-simulated with Bruker WinEPR SimFonia software.

### ESI-MS with Asc

The mass spectra were recorded on a Bruker MicrOTOF-Q spectrometer (Bruker Daltonik, Bremen, Germany), equipped with an Apollo II electrospray ionization source. The following instrumental parameters were set: scanning range 0–2500 *m*/*z*, temperature 200 °C, dry gas-nitrogen, reflector voltage 1300 V, detector voltage 1920 V. The mass spectrometer was operated in positive ion mode. All samples were prepared in water at pH ∼ 7.0. The spectra were measured for the Cu6L system (*C*_L_= 1 mM; 1:1 M:L molar ratio) and the complex with added Asc (*C* = 1 mM) after 1 and 24 h of incubation and infused at a flow rate of 3 μL/min. The instrument was calibrated externally with the Tunemix mixture (Bruker Daltonik, Germany) in the quadratic regression mode.

## ROS Production

### Ascorbic Acid Consumption Test

The UV–vis spectra of ascorbic acid consumption were monitored by the decrease in the maximum absorption band at 265 nm (145 000 M^–1^cm^–1^).^49^ The spectra were recorded as a function of time on a Cary 60 spectrophotometer at 25 °C. All samples were prepared in phosphate-buffered saline (PBS) at pH 6.8 containing ligand (*C* = 12 or 6 μM), Cu­(II) ions in different (1:1, 2:1, and 3:1) M:L molar ratios, and ascorbic acid (*C* = 100 μM) with/without hydrogen peroxide (*C* = 50 μM). Each reaction was repeated three times.

#### Safety Statement

Hydrogen peroxide (H_2_O_2_) was handled with care due to its oxidative and corrosive properties, especially in the concentrated form. Proper PPE was worn, and all manipulations were performed in a fume hood when necessary.

### Luminescence with Terephthalic Acid (TA)

Terephthalic acid (TA), which is a detector of hydroxyl radicals, was used in the study. TA reacts with ^•^OH to form 2-hydroxyterephthalic acid (TAOH). After excitation at 312 nm, an emission peak at 425 nm is observed for TAOH. The increase in the intensity of this band suggests the formation of ^•^OH.^75^ The emission spectra were measured at 1 min intervals for 1 h over the spectral range 300–800 nm and recorded on a Cary Eclipse Fluorescence Spectrophotometer (Agilent Technologies). All samples were prepared in PBS buffer at pH 6.8 containing TA (C = 80 μM), ligand (*C* = 50 μM), Cu­(II) ion in different (1:1, 2:1, and 3:1) M:L molar ratios, ascorbic acid (*C* = 50 μM), hydrogen peroxide (*C* = 50 μM), or their mixture (50 μM/50 μM). The experiment was repeated three times.

#### Safety Statement

Terephthalic acid (TFA) was handled as a fine powder and may cause mild respiratory or skin irritation. Appropriate precautions, such as wearing gloves and lab coats and working in ventilated areas, were taken to minimize exposure.

### EPR Spin Trapping

The identification of free radicals generated by the **Cu6L** system (in a 1:1 M:L molar ratio) in the presence of H_2_O_2_ was carried out by spin trapping experiments. EPR spectrum for control was recorded in the absence of the complex. The 5-dimethyl-1-pyrroline N-oxide (DMPO) was used as a spin trap and applied to the samples immediately before the measurements. The concentrations of the spin trap solution, **Cu6L** system, and H_2_O_2_ were 10 mg/mL, 50 μM, and 25 μM, respectively. The experiment was performed in a glass capillary tube. The spectra were collected at room temperature using a Miniscope MS400 (Magnettech) X-band EPR spectrometer. Typical instrumental settings were microwave frequency 9.43 GHz, scan range 10 mT, sweep time 40 s, time constant 0.1 s, modulation amplitude 0.1 mT, and microwave power 5 mW. The hyperfine coupling constants were obtained by computer simulations with WinEPR SimFonia software.

#### Safety Statement

5,5-Dimethyl-1-pyrroline-*N*-oxide (DMPO), used as a spin trapping agent, may cause irritation to the skin, eyes, and respiratory tract. Although comprehensive toxicological data are limited, all handling was performed with gloves and protective eyewear and waste was disposed of using institutional safety protocols.

### Electrochemical Measurements

Cyclic voltammetry measurements for **5L** and **6L** ligands (1 mM) as well as for their Cu­(II) complexes (**Cu5L** and **Cu6L**) (1:1 M:L molar ratio, [L] = 1 mM) were carried out with the potential control provided by an electrochemical analyzer (Bio-Logic, SP-150). A standard system containing three electrodes was applied: (1) glassy carbon disk as a working electrode (diameter 2 mm), (2) Pt wire as a counter electrode, and (3) reference electrode (Ag/AgCl, 0.22 V vs NHE). All measurements were carried out in PBS buffer solutions using 0.1 M NaNO_3_ as the supporting electrolyte in the selected potential window from −0.2 to 1.0 V vs Ag/AgCl. For each compound, at least three independent measurement cycles were performed with five different scanning rates: 10, 50, 100, 200, 500, and 1000 mV/s.

### Reduction of Cu­(II) Ion by Asc

In the case of the experiment concerning the reduction of the Cu­(II) ion in the complex (*C* = 1 mM) after the addition of ascorbic acid (*C* = 0.25, 0.5, 1, and 5 mM), EPR spectra were measured using a Bruker Elexsys E500 spectrometer operating at a frequency of about 9.6 GHz (X band). The reactions were carried out in sealed glass capillaries. The experimental spectra were simulated by using Bruker WinEPR SimFonia software.

### DNA Damage

The ability of the **Cu6L** system to induce single- or double-strand DNA breaks (final DNA concentration per lane was *C* = 0.31 μg/mL) was investigated with the pBR322 plasmid. All substances were dissolved in PBS buffer (at pH 6.8) with/without hydrogen peroxide (H_2_O_2_, *C* = 50 μM), ascorbic acid (Asc, *C* = 100 μM), potassium iodide (KI, *C* = 8 mM), dimethyl sulfoxide (DMSO, *C* = 0.14 M), or sodium azide (NaN_3_, *C* = 40 mM). After 1 h of incubation at 37 °C, the reaction mixtures (20 μL) were mixed with 3 μL of loading buffer (bromophenol blue in 30% glycerol) and loaded on 1% agarose gel, containing ethidium bromide (EB) in TBE buffer (90 mM Tris borate, 20 mM EDTA, pH 7.4). The concentrations given below the electropherogram are the final ones. The gel electrophoresis was carried out at a constant voltage of 120 V for 120 min. The gel was photographed and processed using a Digital Imaging System (Syngen Biotech). Densitometric analysis was performed using a GelAnalyzer 19.1.

#### Safety Statement

Ethidium bromide (EtBr), a known mutagen, was used in the gel electrophoresis experiment. All handling of EtBr was performed with appropriate safety precautions, including gloves, safety glasses, and lab coats. EtBr handling was performed in designated areas by using appropriate waste disposal procedures in accordance with institutional safety guidelines to minimize exposure and environmental impact. Sodium azide (NaN_3_), a highly toxic and potentially explosive compound, was handled with extreme care. Manipulations were conducted using gloves and eye protection, and all waste was collected separately and disposed of according to hazardous chemical waste guidelines. Dimethyl sulfoxide (DMSO) was used as a solvent. Due to its ability to rapidly penetrate the skin and carry dissolved substances into the bloodstream, all handling was performed with gloves and lab coats to prevent dermal exposure.

### Cell Culture

The mouse colon carcinoma cells (CT26), morphology: fibroblast, ATCC: CRL-2638, were cultured in Dulbecco’s modified Eagle’s medium (DMEM, Diag-Med) with phenol red, supplemented with 10% fetal bovine serum (FBS, Diag-Med) and with 1% streptomycin/penicillin. Cultures were incubated under standard conditions (37 °C and a humidified atmosphere containing 5% CO_2_). Cells were passaged at preconquest density using a solution containing 0.05% trypsin and 0.5 mM EDTA (Sigma-Aldrich).

#### Safety Statement

Cell culture experiments involving murine colon carcinoma cell line CT26 were performed in a certified biosafety cabinet using aseptic techniques in compliance with institutional biosafety protocols. All biological waste was decontaminated prior to disposal.

### MTT Assay

The MTT assay (MTT: 3-(4,5-dimethylthiazol-2-yl)-2,5-diphenyltetrazolium bromide, Sigma-Aldrich) was carried out as described previously.^23^ Ten thousand cells in 200 μL of growth media were seeded in the wells of a 96-well plate. After 24 h, 200 μL of various concentrations of **5L**, **6L**, **Cu5L**, **Cu6L**, and Cu­(II) (0.1, 0.05, 0.01, and 0.001 mM) were added and incubated for 24 h at 37 °C in a CO_2_ incubator. The compounds were dissolved in the respective medium with 1% FBS. Cells were incubated under standard conditions for 24 h, and after that time, the MTT assay was carried out. The surviving fraction was calculated as described elsewhere.^76^ The viability was calculated regarding the untreated cells control. The IC_50_ values were determined using the Hill equation (Origin 9.0).^77,78^ Experiments were carried out in triplicate and repeated at least twice. Determined values of IC_50_ are presented as the mean + SD (standard deviation).

#### Safety Statement

MTT was used for cytotoxicity assays and handled with care due to its potential toxicity and irritant properties.

### Copper Uptake

CT26 cells were seeded in 6-well plates at a density of 500,000 cells per 2 mL and incubated with **Cu5L**, **Cu6L**, and free copper­(II) ions (at the concentration of IC_50_) for 5 min and 4, 12, or 24 h. After appropriate incubation times, the supernatants were removed, and the cells were rinsed with PBS buffer (two times). Then, the cells were harvested from the plate by digesting them with trypsin and washed twice again with PBS buffer. The pellet was mineralized in 65% HNO_3_ (POCh), and the copper concentration was analyzed by mass spectrometry with inductively coupled plasma (ICP-MS) using a PerkinElmer ELAN 6100 facility. The experiment was repeated three times.

### Cellular ROS Production

The generation of ROS and oxidative stress in cells treated with **5L**, **6L**, **Cu5L**, **Cu6L**, and Cu­(II) was monitored by fluorescent probe such as 5-(and-6)-chloromethyl-20,70 dichlorodihydrofluorescein (Sigma-Aldrich), diacetate acetyl ester (H2DCF-DA, Sigma-Aldrich). The influence of various experimental conditions on ROS generation was tested in the separate experiments: (i) changing concentration of the copper compounds (0.1, 0.01, and 0.001 mM), (ii) the addition of N-acetylcysteine (NAC) antioxidant, (iii) the dependence on the incubation time (5 min and 0.5, 4, 12, or 24 h). All experiments were carried out in accordance with the procedures described in our previous papers.^23,27^ Experiments were carried out in triplicate and repeated at least three times.

### MDA Assay

The experiments were performed in 24-well plates, where cells at a density of 50,000 cells per 0.5 mL of medium were seeded. After the medium was removed from the cells, they were washed with PBS buffer. The compounds (**5L**, **6L**, **Cu5L**, **Cu6L**, and H_2_O_2_) at 0.01 mM were added to the cells and incubated for 5 min, 0.5, 4, 6, 12, 24, and 48 h at 37 °C under humidified atmosphere containing 5% CO_2_. The subsequent stages of this analysis were identical to those applied in the experiments without addition of NAC and described elsewhere.^23,27^ Experiments were carried out in triplicate and repeated at least three times.

## Supplementary Material



## Notes

Detailed characterization of the structure of the complexes depending on pH and figures regarding coordination studies, DNA damage, and ROS generation in the cell (PDF)

## Abbreviations

FomA: major outer membrane protein
CRC: colorectal cancer
ROS: reactive oxygen species
6L: Ac-KGHGNGEEGTPTVHNEYH-NH_2_
Asc: ascorbic acid
TAOH: 2-hydroxyterephthalic acid
DMPO: 5,5-dimethyl-1-pyrroline *N*-oxide
DMSO: dimethyl sulfoxide
CT26: colon carcinoma cell line
IC_50_: half-maximal inhibitory concentration
ctrl: control
ctrl1: chymotrypsin-like protease
H2DCF-DA: 2′,7′-dichlorodihydrofluorescein diacetate
DCF: 2′,7′-dichlorofluorescein
MDA: malondialdehyde
NAC: *N*-acetylcysteine
