# Supplementary material for: ’s Copper-FomA Proteins: Driving Cancer Forward
Source: Inorg Chem. 2025 Jul 19;64(30):15502–17. doi: 10.1021/acs.inorgchem.5c01747 (PMC12338124; doi:10.1021/acs.inorgchem.5c01747)
Supplement: Supplementary file 1 [file ic5c01747_si_001.pdf]

Supporting Information

Fusobacterium nucleatum's copper-FomA proteins: driving cancer forward

Monika K. Lesiów[a], Bartosz Kwiatkowski[a], Piotr Pietrzyk[b], Agnieszka Kyzioł[b], Krzysztof Rolka[c], Urszula K. Komarnicka[a]*

[a]Faculty of Chemistry, University of Wrocław, F. Joliot-Curie 14, 50-383 Wrocław, Poland

[b]Faculty of Chemistry, Jagiellonian University, Gronostajowa 2, 30-387 Kraków, Poland

[c]Faculty of Chemistry, University of Gdańsk, Wita Stwosza 63, 80-308 Gdańsk, Poland

corresponding author e-mail: monika.lesiow2@uwr.edu.pl

ABSTRACT

FomA is a major outer membrane protein of *Fusobacterium nucleatum* (*Fn*) overexpressed in the colorectal cancer (CRC) tissues, where increased levels of Cu ions together with reactive oxygen species (ROS) were noted. In this comparative study the connection between *Fn* surrounded by copper ions and the ROS overgeneration leading to the development of CRC is examined. Our study focused on a model peptide Ac-KGHGNGEEGTPTVHNEYH-NH_2 (**6L**) derived from the FomA reflecting a fragment of the protein loop no. 4, exposed to the external environment, which facilitates the binding of Cu ions. The coordination studies (potentiometric titration, UV-Vis, CD, EPR) showed that the **6L** bound Cu(II) and formed mon-, di-, and trinuclear complexes depending on the solution pH. The ability to ROS generation by

promoting the Cu(III)/Cu(II)/Cu(I) redox cycle was proven by CV and EPR techniques. We also confirmed that upon adding Asc, the **6L** was fragmented and after copper coordination, the ROS production increased (e.g.$^{\bullet}OH$, $^1O_2$, $O_2^{\bullet-}$) leading to DNA damage. Stimulation of model mouse colon carcinoma cells by Cu(II) complex with **6L** demonstrated an abundant cellular ROS production, resulted in pronounced lipid peroxidation. Hypothetically, this action mode leads to the damage of colon cells and triggers carcinogenesis processes.

TABLE OF CONTENT

Detailed description of coordination studies..**S-4-S-11**
Table S1. The formation and protonation constants for 6L peptide....................................**S-12**
Table S2. Formation constants and calculated deprotonation constants for mononuclear Cu(II) complexes of Cu6L...**S-13**
Table S3. Spectroscopic parameters for the mono-, di- and trinuclear complexes of Cu6L……………………………………………………………………………**S-14-S-15**
Table S4. Formation constants and calculated deprotonation constants for dinuclear Cu(II) complexes of Cu6L………………………………………………………………………**S-16**
Table S5. Formation constants and calculated deprotonation constants for trinuclear Cu(II) complexes of Cu6L ………………………………………...………………………**S-17**
Fig.S1. EPR spectra of the frozen solution of Cu6L (Cu:L = 1:1, [Cu(II)] = 0.001 M)……**S-18**
Fig.S2. Species distribution diagram of the Cu6L system as a function of pH. Molar ratio Cu: L = 2:1, [Cu(II)] = 0.002 M……………………………………………………………**S-19**
Fig.S3. Electronic absorption spectra for dinuclear complexes of the Cu6L system………**S-20**
Fig.S4. CD spectra in the UV range for dinuclear complexes of the Cu6L system………**S-21**
Fig.S5. EPR spectra of the frozen solution of Cu6L system (Cu:L = 2:1)…………………**S-22**
Fig.S6. Species distribution diagram of the Cu6L system (molar ratio Cu: L = 3:1)……….**S-23**
Fig.S7. Electronic absorption spectra for trinuclear complexes of the Cu6L system………**S-24**
Fig.S8. CD spectra in the UV range for trinuclear complexes of the Cu6L system ………**S-25**
Fig.S9. EPR spectra of the frozen solution of Cu6L system (Cu:L = 3:1)………………….**S-26**
Fig.S10. ESI mass spectrum of the Cu6L (Cu:L = 1:1 molar ratio) in aqueous solution…**S-27**
Fig.S11. ESI mass spectrum of the Cu6L (Cu:L = 2:1 molar ratio) in aqueous solution…**S-28**

Fig.S12. ESI mass spectrum of the Cu6L (Cu:L = 3:1 molar ratio) in aqueous solution…**S-29**
Fig.S13. Percentage of individual DNA form created after adding different Cu(II) complexes to DNA…………………………………………………………………………..**S-29**
Fig.S14. Reduction of fluorescence intensity of CT26 cells incubated with 5L and Cu5L at different concentrations after 5 min of incubation…………………………………………**S-30**
Fig.S15. Reduction of fluorescence intensity of CT26 cells incubated with 5L and Cu5L at different concentrations after 30 min of incubation………………………………………**S-30**
Fig.S16. Reduction of fluorescence intensity of CT26 cells incubated with 5L and Cu5L at different concentrations after 4 h of incubation……………………………………………**S-31**
Fig.S17. Reduction of fluorescence intensity of CT26 cells incubated with 5L and Cu5L at different concentrations after 12 h of incubation…………………………………………**S-31**
Fig.S18. Reduction of fluorescence intensity of CT26 cells incubated with 5L and Cu5L at different concentrations after 24 h of incubation…………………………………………**S-32**
Figure S19. Reduction of fluorescence intensity of CT26 cells incubated with 6L and Cu6L at increasing incubation time………………………………………………………………**S-32**

Coordination studies

Protonation constants of studied ligand

The Ac-KGHGNGEEGTPTVHNEYH-NH_2 ligand (**6L**) behaves like octaprotic acid (H_8L) in aqueous solution. This means that this peptide exhibits eight protonation sites that can be assigned to: three carboxyl groups from the side chain of the glutamic acid (Glu) residues, three imidazole groups from side chain of the histidyl residues (His), the phenolic group of the tyrosine residue (Tyr) and the amino group of the lysyl residue (Lys). The formation (logβ) and protonation constants (log$K$) of the **6L** ligand obtained by theoretical simulation of the potentiometric data are summarized in **Table S1**.

The calculated average protonation constant values (log$K$) for the -COO- groups from the side chain of the Glu residues for **6L** peptide equals 4.06. This value complies with those reported in the literature and is in agreement with the average log$K$ values obtained for the same groups belonging to the Ac-KGHGNGEEGTPTVHNE-NH_2 (**3L**) ligand and its cyclic counterpart cyclo (KGHGNGEEGTPTVHNE) (**4L**) being also fragments of the FomA protein.[1] A slight difference between the three log$K$ values for the carboxyl groups of the Glu residues (0.52- 0.66 log units) suggest that the protonation processes of these groups overlap. The calculated constants are macro constants and refer to the protonation of three carboxyl groups from Glu residues and cannot be assigned to specific Glu residues.

The lowest protonation constant (log$K$ = 3.45) (significantly different from the others) is attributed to the carboxyl group of the side chain of Glu residue located at the C-terminus of the peptide chain (**Table S1**).[2] The calculated average values of the next three protonation constants (log$K$ = 6.62) corresponds to the protonation of the pyridine nitrogen of the imidazole rings. Analogous log$K$ values are reported in the literature for the peptides including three His residues in their sequence[3] as well as for the **3L** and **4L** fragments from FomA protein mentioned above. The differences between the three protonation constants for the His residues of **6L** ligand are rather small (0.52-0.62 log unit) and suggest the simultaneous protonation of the pyridine nitrogen atoms of His residues. Thus, it is impossible to unambiguously assign the log$K$ value to the imidazole nitrogen atom of a specific His residue.

The highest log$K$ values refer to the phenolic group (-OH) of the side chain of the Tyr residue (log$K$ = 9.49) and to the amino group (-NH_2) of the side chain of the Lys residue (log$K$ = 10.32). The obtained log$K$ values are comparable with those described in the literature and

with values of those obtained for the groups from different ligands from FomA protein.[4, 5] They are consistent with those obtained for the protonation of the (-O-) group of the PTVHNEYH-NH_2 (**5L**) ligand and the attachment of the proton to the -NH_2 group of the side chain of the Lys residue of Ac-KGHGNG-NH_2 (**1L**), Ac-KGHGNGEEGTPTVHNE-NH_2 (**3L**) and cyclo (KGHGNGEEGTPTVHNE) (**4L**).

Mononuclear copper(II) complexes

The formation ($\log\beta$) and deprotonation constants (pK_a) for the Cu(II)-Ac-KG**H**GNGEEGTPTV**H**NEY**H**-NH_2 (**Cu6L**) system were determined by poteniometric data calculations. The presence of three histydyl residues in a peptide sequence enables an independent binding of three Cu(II) ions in the solution what results in the formation of mono- di- and trinuclear complexes. The stability constants calculated from the potentiometric data for the mononuclear complexes are presented in **Table S2**, while the $\log K^*$ values that allowed to assess the affinity of the peptides to the metal ions are collected in **Table S3**.[6, 7] The spectroscopic methods such as UV-Vis, CD and EPR were used in order to verify the computational models. The ESI-MS technique confirmed the possibility of forming complexes with a given stoichiometry determined on the basis of the calculations of potentiometric data but in the gas phase.

The coordination process of Cu(II) ions to the ligand (1:1 M:L molar ratio) was monitored in the pH range from 2.5 to 10.5 with particular emphasis on coordination modes in pH for the proximal (right; pH range 5.5 – 7.5) and distal (left; 6.5 – 7.5)) parts of the large intestine.[8] The gradual deprotonation of the ligand and the subsequent coordination of the Cu(II) ion led to the formation of nine complexes in the solution: CuH_5L, CuH_4L, CuH_3L, CuH_2L, CuHL, CuL, $CuH_{-1}L$, $CuH_{-2}L$ and $CuH_{-3}L$. The general formula of the complexes is CuH_nL, where n ranges from +5 to -3 (**Table S2**, **Fig.2A**). The formation of dinuclear complexes in the low concentrations (representing from 5% to 25% of the aqueous solution in the pH range from 4.5 to 10.5) was also observed.

At pH 3.0, the dissociation of three protons from the ligand occurs and the first CuH_5L species is formed. The stoichiometry of this complex indicates that the Cu(II) ion is bound by the ligand via the imidazole nitrogen atom of the His residue (**Table S2**). The spectroscopic characteristic of the CuH_5L species is not possible, due to its low concentration in an aqueous solution (less than 20% at the maximum of occurrence), as well as overlapping with the aqua Cu(II) complex and other species (**Fig.2A**).

As the pH values increases, the two new species of CuH_4L and CuH_3L stoichiometry with $pK_{a(5/4)} = 4.44$ and $pK_{a(4/3)} = 4.30$ are formed. A slight difference (less than 0.6 log units) between these two values of deprotonation constants indicates simultaneous deprotonation processes. The low abundance of the CuH_4L species (with a maximum prevalence of less than 20% of the solution) and the coexistence with the aqua Cu(II) complex and other species made it impossible to determine the coordination mode based on spectroscopic analysis. However, the $pK_{a(5/4)}$ value for the formation of the CuH_4L complex can be assigned to the proton dissociation from the carboxyl group of a glutamic acid (Glu) residue.

The CuH_3L species dominates at pH 5.2, where it constitutes about 60% of the solution (**Fig.2A**). The calculated $\log K^*$ value equal to -5.31 for the CuH_3L complex is associated with deprotonation and coordination of the second imidazole nitrogen atom from the histidyl residue.[9] Moreover, the obtained $\log K^*$ value is similar to those calculated for the **Cu3L**, **Cu4L** and **Cu5L** systems involving the same donor atoms in the coordination plane. However, it is more than one order of magnitude greater than the $\log K^*$ value calculated for the analogous coordination mode of the Cu(II)-Ac-EVHHQKLVFF-NH_2 described in the literature.[10] This difference may indicate the existence of additional interactions of amino acid residues in the CuH_3L complex of the Cu(II)-Ac-KGHGNGEEGTPTVHNEYH-NH_2 system, which contribute to a significant stabilization of the complex. The *d-d* transition band at 671 nm in the UV-Vis spectra (691 nm based on the Prenesti equation) confirms the participation of the second nitrogen atom from the histidyl residue in the coordination process (**Fig.2B**, **Table S3**). The experimental value is lower than expected what may indicate additional interactions of the ligand with the Cu(II) ion.[11, 12] The EPR parameters ($A_{||} = 16.7$ mT and $g_{||} = 2.297$) also prove existence of the 2N $\{2N_{im}\}$ donor set (**Table S3**, **Fig.S1A**).[12, 13]

A further increase in the pH of the solution contributes to the deprotonation of the second and the third amide nitrogen ($pK_{a(1/0)} = 7.92$ and $pK_{a(0/-1)} = 7.76$) and the formation of CuL and $CuH_{-1}L$ complexes, respectively (**Table S2**).[14, 15] The spectroscopic analysis of the CuL complex is impossible due to the low prevalence of the CuL species in the solution (with the maximum presence of about 20% of the solution) and coexistence with other CuHL and $CuH_{-1}L$ complexes (**Fig.2A**). However, the stoichiometry of the CuL species allows to propose 4N $\{2N_{im}, 2N^-\}$ donor set. The calculated $\log K^*$ value (-20.80) for $CuH_{-1}L$ species confirms the existence of 4N $\{N_{im}, 3N^-\}$ coordination mode.[3] This complex indicates comparable stability to the 4N complexes with FomA protein fragments containing two histidyl residues in their sequences. On the other hand, the presence of three His residues in the studied ligand molecule (**6L**) increases the stability of the $CuH_{-1}L$ complex with the $\{N_{im}, 3N^-\}$ coordination mode

compared to the analogous 4N system with FomA protein fragments containing two adjacent (Ac-NHHTLND-NH_2; **7L**) or one (Ac-KGHGNG-NH_2; **1L**, Ac-PTVHNE-NH_2; **2L**) histidyl residues.[16, 17] It should also be noted that this species is much more stable (more than three logarithmic units) than the corresponding systems described in the literature for Cu(II)-Ac-HGGGWGQ-NH_2[18] or Cu(II)-Ac-EVHHQK-NH_2.[19] The 4N {N_{im}, $3N^-$} binding mode proposed for the $CuH_{-1}L$ system was also confirmed by spectroscopic methods. The UV-Vis spectrum shows the *d-d* transition band at 559 nm (value predicted by the Prenesti method is 523 nm) (**Table S3**, **Fig.2B**).[20] The difference in the position of the maximum absorption of the band may be caused by the interaction of amino acid residues of the ligand (*e.g.* Glu) with Cu(II) ions. Moreover, the CD spectrum shows two CT bands occurring at 255 nm ($N_{Im}\pi_2 \rightarrow$ Cu(II)) and at 328 nm ($N^-_{(amide)} \rightarrow$ Cu (II) and $N_{Im}\pi_1 \rightarrow$ Cu(II)) (**Table S3**, **Fig.2C**).[12, 21] The presence of four nitrogen atoms in the equatorial plane around Cu(II) ions is also confirmed by the determined EPR parameters ($A_{||}$ = 17.9 mT, $g_{||}$ = 2.227, $g_{\perp}$ = 2.053) (**Table S3**, **Fig.S1D**).[14] They also prove the existence of a complex with axial symmetry.

Further deprotonation of the $CuH_{-1}L$ species and the formation of the $CuH_{-2}L$ complex is related to the dissociation of the phenolic group (-OH) of the side chain of the tyrosyl (Tyr) residue. The phenol group of the Tyr residue is not involved in the coordination process, despite the lower $pK_{a(-1/-2)}$ = 9.18 compared to the (log*K* = 9.49) for the free ligand.[22]

Analogous spectroscopic parameters as for the $CuH_{-2}L$ complex were obtained for the next species of $CuH_{-3}L$ (**Table S3**). The formation of $CuH_{-3}L$ complex is the result of deprotonation ($pK_{a(-2/-3)}$ = 10.15) of the ε-amino group of the Lys residue, which does not take part in the coordination process of Cu(II) ions.[23] The involvement of four nitrogen atoms in the metal ion coordination process in the $CuH_{-3}L$ complex was proved by the EPR parameters ($A_{||}$ = 19. 9 mT, ${}^{Cu}A_{\perp}$ = 2.65 mT, ${}^{N}A_{\perp}$ = 1.61 mT, $g_{||}$ = 2.192, $g_{\perp}$ = 2.046) and the registration of 9 resonance lines resulting from hyperfine interaction between the spin of the unpaired Cu(II) electron and the total nuclear spin of coordinated nitrogen atoms (I^{14}N = 1) (**Table S3**, **Fig.S1F**).

Dinuclear copper(II) complexes

The distribution diagram of the species for the studied system in the 2:1 M:L molar ratio as a function of pH is shown in **Figure S2**. The binding of Cu(II) ions to the ligand was monitored in the pH range from 2.5 to 10.5. The analysis of the data obtained from potentiometric titration confirmed that the studied peptide after binding the Cu(II) ion forms mononuclear complexes (in the pH range from 3.0 to 8.0), trinuclear systems (at very low concentrations, from pH 5.5 to 10.5) and six dinuclear species: Cu_2HL, $Cu_2H_{-2}L$, $Cu_2H_{-3}L$,

$Cu_2H_{-4}L$, $Cu_2H_{-5}L$ and $Cu_2H_{-6}L$ (in the pH range from 4.5 to 10.5) in the aqueous solution (**Fig. S2**, **Table S4**).

The first dinuclear complex formed in aqueous solution is the Cu_2HL, which is about 45% of the solution at pH 6.0. The stoichiometry of this species suggested the 2N $\{N_{im}, N^-\}$ 2N $\{2N_{im}\}$ coordination mode, which is also confirmed by the presence of the *d-d* band on the UV-Vis spectrum at 639 nm (**Fig.S3**) and the charge transfer transition on the CD spectrum ($N_{Im\pi2} \rightarrow$ Cu(II) at 259 nm) (**Table S3**, **Fig.S4**).[11, 12]

The formation of the $Cu_2H_{-3}L$ species ($pK_{a(-2/-3)} = 7.51$) above pH 6.5 may be associated with the gradual deprotonation and coordination of the next amide nitrogen atom with the formation of the 4N $\{N_{im}, 3N^-\}$ 4N $\{2N_{im}, 2N^-\}$ coordination mode (**Table S4**, **Fig.S2**).[24] However, the spectroscopic characteristic of this species is not possible due to its coexistence with the $Cu_2H_{-2}L$ and $Cu_2H_{-4}L$ complexes in the solution (**Fig.S2**).

As the pH of the solution increases, the $Cu_2H_{-4}L$ species (constituting ~ 50% of the solution at pH 8.7) is formed (**Fig.S2**). The determined deprotonation constant $pK_{a(-3/-4)} = 8.23$ for $Cu_2H_{-4}L$ complex indicates the involvement of the sixth amide nitrogen atom in the Cu(II) coordination process.[25] The *d-d* transition band on the UV-Vis spectrum at 572 nm and the charge transfer transition on the CD spectrum ($N_{Im\pi2} \rightarrow$ Cu(II) at 252 nm, $N^-_{(amide)} \rightarrow$ Cu(II) and $N_{Im\pi1} \rightarrow$ Cu(II) at 326 nm) suggest that Cu(II) ions in the $Cu_2H_{-4}L$ complex are bound by four nitrogen atoms forming a 4N $\{N_{im}, 3N^-\}$ 4N $\{N_{im}, 3N^-\}$ coordination mode (**Table S3**, **Figs.S3**, **S4**).[12, 21] Two *d-d* transitions occurring in the visible range of the CD spectrum at 550 and 649 nm indicate changes in the equatorial plane of Cu(II) ions (**Table S3**). The EPR parameters ($A_{||} = 17.5$ mT, $g_{||} = 2.230$) also confirmed the proposed binding sites in the $Cu_2H_{-4}L$ complex (**Table S3**, **Fig.S5A**).[14]

The formation of the $Cu_2H_{-5}L$ species above pH 8.0 is probably the result of deprotonation of the phenolic group (-OH) of the tyrosyl residue side chain. It is evidenced by the obtained $pK_{a(-4/-5)} = 9.19$ (**Table S4**).[1] However, the phenolic group does not take part in the metal ion binding process. Determination of spectroscopic parameters for the $Cu_2H_{-5}L$ complex turned out to be impossible due to its coexistence with the $Cu_2H_{-4}L$ and $Cu_2H_{-6}L$ species in the solution (**Fig.S2**).

Above pH 8.5, the last complex ($Cu_2H_{-6}L$) with $pK_{a(-5/-6)} = 9.56$) is formed in aqueous solution (**Fig.S2**). This deprotonation step is associated with the loss of a proton from the ε-amino group of the lysyl residue, which not participate in coordination of Cu(II) ions.[1] Based on the spectroscopic parameters: $\lambda_{max} = 537$ nm obtained from the UV-Vis spectrum (**Fig.S3**), wavelengths for CT transitions on the CD spectrum ($N_{Im\pi2} \rightarrow$ Cu(II) at 252 nm, $N^-_{(amide)} \rightarrow$

Cu(II) and $N_{Im\pi1}$ → Cu(II) at 316 nm) (**Fig.S4**) and $A_{||}$ = 18.0 mT, $g_{||}$ = 2.228 (obtained from simulation of the EPR spectrum, **Fig.S5C**) for the $Cu_2H_{-6}L$ species, the following 4N {N_{im}, $3N^-$} 4N {N_{im}, $3N^-$} coordination mode was proposed. The same donor atoms as for the $Cu_2H_{-6}L$ species were also noted for the previous $Cu_2H_{-4}L$ and $Cu_2H_{-5}L$ complexes (**Table S3**).[11, 21, 26]

Trinuclear copper(II) complexes

The presence of three binding sites (three histidyl residues: His^3, His^{14} and His^{18}) in the studied peptide enables the independent coordination of Cu(II) ions and formation of trinuclear complexes. The distribution diagram of complexes (in the 3:1 M:L molar ratio) is shown in **Figure S6**. The formation of nine different trinuclear species: $Cu_3H_{-2}L$, $Cu_3H_{-3}L$, $Cu_3H_{-4}L$, $Cu_3H_{-5}L$, $Cu_3H_{-6}L$, $Cu_3H_{-7}L$, $Cu_3H_{-8}L$, $Cu_3H_{-9}L$ and $Cu_3H_{-10}L$ in the pH range from 5.5-10.5 is observed (**Fig.S6**). The mononuclear Cu(II) complexes are also present in the solution in the pH range from 3.0 to 6.5, while the dinuclear complexes in the pH range from 4.5 to 7.5 (**Fig.S6**).

The first trinuclear species ($Cu_3H_{-2}L$) starts to form above pH 5.5 (about 40% at pH 6.4) (**Fig.S6**). Based on the UV-Vis (λ_{max} = 600 nm corresponding to the *d-d* transition band) (**Fig.S7**) and CD parameters (λ = 251 nm for the $N_{Im\pi2}$ → Cu(II), λ = 310 nm for $N^-_{(amide)}$ → Cu(II) and λ = 356 nm for $N_{Im\pi1}$ → Cu(II) charge transfer transitions in the ultraviolet range (**Fig.S8**) and λ = 514 nm and 601 nm for the *d-d* bands in the visible range) the following 3N {N_{im}, $2N^-$} 2N {N_{im}, N^-} 2N {N_{im}, N^-} coordination mode for $Cu_3H_{-2}L$ species was proposed (**Table S3**).[11, 24, 27]

The increase in the pH value leads to a gradual dissociation of the $Cu_3H_{-2}L$ species and formation of the $Cu_3H_{-3}L$ ($pK_{a(-2/-3)}$ = 6.78) and then $Cu_3H_{-4}L$ complexes ($pK_{a(-3/-4)}$ = 6.57) (**Table S5**, **Fig.S6**). A slight difference between these two pK_a values (less than 0.6 log unit) suggests that both deprotonation processes run simultaneously. The obtained pK_a value for $Cu_3H_{-3}L$ species confirm the deprotonation and coordination of the fifth amide nitrogen atom.[27] However, spectroscopic characteristics of this species is not possible, due to the too low concentration of the complex in the aqueous solution (about 20% at its maximum prevalence) and the coexistence with other species present in the solution (**Fig.S6**). Therefore, based on the stoichiometry of the $Cu_3H_{-3}L$ complex, the 3N {N_{im}, $2N^-$} 3N {N_{im}, $2N^-$} 2N {N_{im}, N^-} coordination mode can be proposed.

Above pH 6.0, the $Cu_3H_{-5}L$ species with the $pK_{a(-4/-5)}$ = 7.06 is formed. The pK_a value indicates the involvement of next amide nitrogen atom in the metal ion coordination process (**Table S5**).[25] This species constitutes about 65% of the solution at pH 7.7 (**Fig.S6**). Determined spectroscopic parameters: λ_{max} = 592 nm for the *d-d* electron transition on the UV-Vis spectrum,

position of the charge transfer bands on the CD spectrum ($N_{Im\pi2}$ → Cu(II) at 251 nm, $N^{-}_{(amide)}$ → Cu(II) and $N_{Im\pi1}$ → Cu(II) at 355 nm), and $A_{||}$ = 17.1 mT, $g_{||}$ = 2.233 (obtained from simulation of the EPR spectrum) allowed to assign species the 4N {N_{im}, $3N^-$} 3N {N_{im}, $2N^-$} 3N {N_{im}, $2N^-$} coordination mode to the $Cu_3H_{-5}L$ complex (**Table S3**, **Figs.S7-S9A**).[11, 21, 24, 27]

The formation of $Cu_3H_{-6}L$ and $Cu_3H_{-7}L$ complexes is associated with the gradual deprotonation and the subsequent coordination process of amide nitrogen atoms with $pK_{a(-5/-6)}$ = 8.23 and $pK_{a(-6/-7)}$ = 8.85, respectively (**Table S5**).[9] In the $Cu_3H_{-6}L$ complex, two metal ions are bound by four nitrogen atoms 4N {N_{im}, $3N^-$} 4N {N_{im}, $3N^-$}, while the third Cu(II) ion is coordinated by three nitrogen atoms 3N {N_{im}, $2N^-$}. In turn, in the $Cu_3H_{-7}L$ species, the three Cu(II) ions are surrounded by 4N {N_{im}, $3N^-$} donor set. However, it was not possible to determine the spectroscopic parameters for both complexes due to their coexistence with other species present in the aqueous solution (**Fig.S6**).

The $Cu_3H_{-8}L$ species dominates at pH 9.6 and constitutes about 55% of the solution) (**Fig.S6**). Its presence is associated with deprotonation of the -OH group from the Tyr residue ($pK_{a(-7/-8)}$ = 9.11), which does not coordinate to Cu(II) ion (**Table S5**).[22] The following 4N {N_{im}, $3N^-$} 4N {N_{im}, $3N^-$} 4N {N_{im}, $3N^-$} coordination mode for the $Cu_3H_{-8}L$ species was proposed based on UV-Vis (λ_{max} = 553 nm for the d-d transition), CD (location of the charge transfer bands: $N_{Im\pi2}$ → Cu(II) at 251 nm, $N^{-}_{(amide)}$ → Cu(II) and $N_{Im\pi1}$ → Cu(II) at 319 nm) and EPR studies ($A_{||}$ = 16.8 mT, $g_{||}$ = 2.248) (**Table S3**, **Figs.S7-S9B**).[11, 21, 24, 27] The relatively low energy of the d-d transition in the UV-Vis spectrum, as well as the lower value of the EPR parameters obtained may indicate slight disturbances in the symmetry of the $Cu_3H_{-8}L$ complex.

The $Cu_3H_{-9}L$ species is the next one formed in solution with a $pK_{a(-8/-9)}$ value of 10.10 (**Table S5**). This deprotonation is associated with the loss of a proton from the ε-amino group of the lysyl residue.[5] However, this moiety is not involved in the coordination process although the value of the deprotonation constant for the $-NH_2$ group of the Lys residue from the $Cu_3H_{-9}L$ complex is lower than the log$K$ value for this group in the ligand (log$K$ = 10.32) (**Tables S1, S5**). The spectroscopic characteristics of the $Cu_3H_{-9}L$ complex is not possible due to its low concentration in the solution, as well as its coexistence with other species ($Cu_3H_{-8}L$ and $Cu_3H_{-10}L$) (**Fig.S6**). Therefore, based on the stoichiometry of the complex and potentiometric data, the 4N {N_{im}, $3N^-$} 4N {N_{im}, $3N^-$} 4N {N_{im}, $3N^-$} set of donor atoms for $Cu_3H_{-9}L$ was proposed.

The last step of the conversion of $Cu_3H_{-9}L$ to $Cu_3H_{-10}L$ species with $pK_{a(-9/-10)}$ = 9.98 is probably associated with the deprotonation of the N(1)H group of the imidazole ring from the histidyl residue coordinated to the Cu(II) ion. This process takes place simultaneously with the earlier dissociation reaction of the $Cu_3H_{-8}L$ to $Cu_3H_{-9}L$ species (the difference between the two

pK_a values is less than 0.6 log units) (**Table S5**).[25] However, the final deprotonation process does not affect the coordination of the metal ion. The lack of significant changes in the spectroscopic parameters for the $Cu_3H_{-10}L$ species (compared to the $Cu_3H_{-8}L$ complex) allows this species to be assigned the same donor atoms as for the $Cu_3H_{-7}L$, $Cu_3H_{-8}L$ and $Cu_3H_{-9}L$ complexes (**Table S3**).

The ESI-MS method was used to support the stoichiometry of the complexes, the formation of which was recorded in the solution. ESI-MS spectra for the Cu6L system in the 1:1 M:L molar ratio were recorded in the positive ionization mode. The peaks of the following mononuclear ions of the complexes were detected: $CuHL^{3+}$ (*m/z* 689.6 Da) (**Fig.S10**), CuL^{2+} (*m/z* 1033.9 Da) and $CuH_{-1}L^{+}$ (*m/z* 2066.9 Da). In the ESI-MS experimental conditions in the 1:1 M:L molar ratio the formation of dinuclear complexes of Cu(II) was also observed. The occurring of the following signals of the $Cu_2H_{-1}L^{3+}$ (*m/z* 709.9 Da) and $Cu_2H_{-2}L^{2+}$ (*m/z* 1064.4 Da) ions was noticed.

The ESI-MS spectra measured in the positive ionization mode for the studied system in the 2:1 molar ratio show the dinuclear ion peaks of the Cu(II) complexes: $Cu_2H_{-1}L^{3+}$ (*m/z* 709.9 Da) and $Cu_2H_{-2}L^{2+}$ (*m/z* 1064.4 Da) (**Fig.S11**).

In turn, the ESI-MS spectra recorded in the positive ion mode for the system in the 3:1 M:L molar ratio showed the presence of peaks of trinuclear Cu(II) complexes: $Cu_3H_{-3}L^{3+}$ (*m/z* 730.2 Da) and $Cu_3H_{-4}L^{2+}$ (*m/z* 1094.8 Da) (**Fig.S12**).

Table S1. The formation (logβ) and protonation constants (log$K$) for **6L** peptide (I = 0.1 M KNO_3, T = 298 K).

Species	6L
**logβ***	
**HL**	10.32(1)
**H_2L**	19.81(1)
**H_3L**	27.02(1)
**H_4L**	33.61(1)
**H_5L**	39.68(1)
**H_6L**	44.31(1)
**H_7L**	48.42(1)
**H_8L**	51.87(1)
**log$K$**	
**NH_2 (Lys)**	10.32
**O^- (Tyr)**	9.49
**His**	7.21
**His**	6.59
**His**	6.07
**COO^- (Glu)**	4.63
**COO^- (Glu)**	4.11
**COO^- (Glu)**	3.45

*Formation constants (logβ) and protonation constants (log$K$) for ligand are expressed by the equations: $\beta(H_nL)=[H_nL]/[L][H^+]^n$ and $\log K=\log\beta(H_nL)-\log\beta(H_{n-1}L)$, respectively. Standard deviations (3σ values) on the last digit of formation and protonation constants are given in parentheses. Charges are omitted for clarity.

Table S2. Formation constants (log*β*) and calculated deprotonation constants (pK_a) for mononuclear Cu(II) complexes of **Cu6L** (*I* = 0.1 M KNO_3, *T* = 298 K).

**Species**	**Cu6L**
	**log*β****
**CuH_5L**	43.11(4)
**CuH_4L**	38.67(4)
**CuH_3L**	34.37(2)
**CuH_2L**	28.72(2)
**CuHL**	21.90(3)
**CuL**	13.98(6)
**$CuH_{-1}L$**	6.22(4)
**$CuH_{-2}L$**	-2.96(4)
**$CuH_{-3}L$**	-13.11(5)
	**pK_a***
**p$K_{(5/4)}$**	4.44
**p$K_{(4/3)}$**	4.30
**p$K_{(3/2)}$**	5.65
**p$K_{(2/1)}$**	6.82
**p$K_{(1/0)}$**	7.92
**p$K_{(0/-1)}$**	7.76
**p$K_{(-1/-2)}$**	9.18
**p$K_{(-2/-3)}$**	10.15

*Formation constants (log*β*) and deprotonation constants (pK_a) for the Cu6L system are expressed by the following equations: $\beta(CuH_nL)=[CuH_nL]/[Cu][L][H^+]^n$ and $pK_a=\log\beta(CuH_nL)-\log\beta(CuH_{n-1}L)$, respectively. Standard deviations (3σ values) on the last digit of formation and protonation constants are given in parentheses. Charges are omitted for clarity.

Table S3. Spectroscopic parameters for the mono-, di- and trinuclear complexes of **Cu6L**.

Species	pH	UV-Vis		CD		EPR				
		λ [nm]	ε [M^{-1}cm^{-1}]	λ [nm]	$\Delta\varepsilon$ [M^{-1}cm^{-1}]	$g_{\|\|}$ (g_{zz})	$g_{\perp}$ (g_{xx}, g_{yy})	$^{Cu}A_{\|\|}$ [mT]	$^{Cu}A_{\perp}$ [mT]	$^{N}A_{\perp}$ [mT]
**Cu6L 1:1**										
CuH_3L<br>**2N {2N$_{im}$}**	5.2	671[a]	27	-	-	2.297	2.068	16.7	unresolved	unresolved
CuH_2L<br>**3N {3N$_{im}$}**	6.2	635[a]	38	-	-	2.282	2.066	17.0	unresolved	unresolved
CuHL<br>**4N {3N$_{im}$, N$^-$}**	7.2	591[a]	45	256[b]<br>307[c]<br>347[d]	1.88<br>-0.24<br>0.10	2.267	2.060	17.4	unresolved	unresolved
$CuH_{-1}L$<br>**4N {N$_{im}$, 3N$^-$}**	8.6	559[a]	52	255[b]<br>328[c,d]<br>548[a]<br>654[a]	2.99<br>0.16<br>-0.27<br>0.14	2.227	2.053	17.9	unresolved	unresolved
$CuH_{-2}L$<br>**4N {N$_{im}$, 3N$^-$}**	9.7	539[a]	62	255[b]<br>319[c,d]<br>534[a]<br>647[a]	2.96<br>0.45<br>-0.56<br>0.32	2.207	2.046	19.7	unresolved	unresolved
$CuH_{-3}L$<br>**4 N {N$_{im}$, 3 N$^-$}**	10.5	536[a]	66	254[b]<br>317[c]<br>533[a]<br>645[a]	3.17<br>0.61<br>-0.66<br>0.37	2.192	2.046	19.9	2.65	1.61
**Cu6L 2:1**										
Cu_2HL<br>**2N {N$_{im}$, N$^-$}**<br>**2N {2N$_{im}$}**	6.0	639[a]	48	259[b]	0.74	-	-	-	-	-
$Cu_2H_{-2}L$<br>**3N {N$_{im}$, 2N$^-$}**	7.1	595[a]	78	252[b]<br>307[c]	4.84<br>-0.31	2.230	2.061;<br>2.066	17.2	-	-

**4N {2N_{im}, 2N^-}**<br>lub<br>**4N {N_{im}, 3N^-}**<br>**3N {2N_{im}, N^-}**				357[d]<br>514[a]<br>598[a]	0.26<br>0.18<br>-0.17					
$Cu_2H_{-4}L$<br>**4N {N_{im}, 3N^-}**<br>**4N {N_{im}, 3N^-}**	8.7	572[a]	90	252[b]<br>326[c,d]<br>550[a]<br>649[a]	5.17<br>0.28<br>-0.27<br>0.18	2.230	2.068	17.5	**-**	**-**
$Cu_2H_{-6}L$<br>**4N {N_{im}, 3N^-}**<br>**4N {N_{im}, 3N^-}**	10.5	537[a]	117	252[b]<br>316[c,d]<br>524[a]<br>637[a]	5.68<br>1.36<br>-1.05<br>0.69	2.228	2.044	18.0	**-**	**-**
**Cu6L 3:1**										
$Cu_3H_{-2}L$<br>**3N {N_{im}, 2N^-}**<br>**2N {N_{im}, N^-}**<br>**2N {N_{im}, N^-}**	6.4	600[a]	89	251[b]<br>310[c]<br>356[d]<br>514[a]<br>601[a]	4.72<br>-0.14<br>0.39<br>0.18<br>-0.19	-	-	-	**-**	**-**
$Cu_3H_{-5}L$<br>**4N {N_{im}, 3N^-}**<br>**3N {N_{im}, 2N^-}**<br>**3N {N_{im}, 2N^-}**	7.7	592[a]	117	251[b]<br>355[c,d]<br>505[a]<br>574[a]	6.25<br>0.35<br>0.13<br>-0.11	2.233	2.066	17.1	**-**	**-**
$Cu_3H_{-8}L$<br>**4N {N_{im}, 3N^-}**<br>**4N {N_{im}, 3N^-}**<br>**4N {N_{im}, 3N^-}**	9.6	553[a]	139	251[b]<br>319[c,d]<br>526[a]<br>636[a]	5.54<br>1.14<br>-0.83<br>0.56	2.248	2.049	16.8		
$Cu_3H_{-10}L$<br>**4N {N_{im}, 3N^-}**<br>**4N {N_{im}, 3N^-}**<br>**4N {N_{im}, 3N^-}**	10.5	543[a]	158	251[b]<br>317[c,d]<br>525[a]<br>636[a]	6.34<br>1.63<br>-1.21<br>0.72	2.248	2.051	18.4		

[a]*d-d* transition; [b]$N_{Im}(\pi_1)\rightarrow$Cu(II) charge transfer transition; [c]$N_{Im}(\pi_2)\rightarrow$Cu(II) charge transfer transition; [d]$N^{-}_{(amid)}\rightarrow$Cu(II) charge transfer transition.

Table S4. Formation constants (logβ) and calculated deprotonation constants (pK_a) for dinuclear Cu(II) complexes of **Cu6L** (I = 0.1 M KNO_3, T = 298 K).

**Species**	**Cu6L**
	**logβ***
**Cu_2HL**	26.27(2)
**$Cu_2H_{-2}L$**	6.87(4)
**$Cu_2H_{-3}L$**	-0.64(3)
**$Cu_2H_{-4}L$**	-8.87(4)
**$Cu_2H_{-5}L$**	-18.06(4)
**$Cu_2H_{-6}L$**	-27.62(3)
	**pK_a***
**p$K_{(-2/-3)}$**	7.51
**p$K_{(-3/-4)}$**	8.23
**p$K_{(-4/-5)}$**	9.19
**p$K_{(-5/-6)}$**	9.56

*Formation constants (logβ) and deprotonation constants (pK_a) for the Cu6L system are expressed by the following equations: $\beta(Cu_2H_nL)=[Cu_2H_nL]/[Cu]^2[L][H^+]^n$ and $pK_a=\log\beta(Cu_2H_nL)-\log\beta(Cu_2H_{n-1}L)$, respectively. Standard deviations (3σ values) on the last digit of formation and protonation constants are given in parentheses. Charges are omitted for clarity.

Table S5. Formation constants (logβ) and calculated deprotonation constants (pK_a) for trinuclear Cu(II) complexes of **Cu6L** (I = 0.1 M KNO_3, T = 298 K).

**Species**	**Cu6L**
	**logβ***
**$Cu_3H_{-2}L$**	10.78(5)
**$Cu_3H_{-3}L$**	4.00(1)
**$Cu_3H_{-4}L$**	-2.57(7)
**$Cu_3H_{-5}L$**	-9.63(4)
**$Cu_3H_{-6}L$**	-17.86(5)
**$Cu_3H_{-7}L$**	-26.71(6)
**$Cu_3H_{-8}L$**	-35.82(5)
**$Cu_3H_{-9}L$**	-45.92(9)
**$Cu_3H_{-10}L$**	-55.90(5)
	**pK_a***
**p$K_{(-2/-3)}$**	6.78
**p$K_{(-3/-4)}$**	6.57
**p$K_{(-4/-5)}$**	7.06
**p$K_{(-5/-6)}$**	8.23
**p$K_{(-6/-7)}$**	8.85
**p$K_{(-7/-8)}$**	9.11
**p$K_{(-8/-9)}$**	10.10
**p$K_{(-9/-10)}$**	9.98

*Formation constants (logβ) and deprotonation constants (pK_a) for the Cu6L system are expressed by the following equations: $\beta(Cu_3H_nL)=[Cu_3H_nL]/[Cu]^3[L][H^+]^n$ and pK_a=log$\beta(Cu_3H_nL)$-log$\beta(Cu_3H_{n-1}L)$, respectively. Standard deviations (3σ values) on the last digit of formation and protonation constants are given in parentheses. Charges are omitted for clarity.

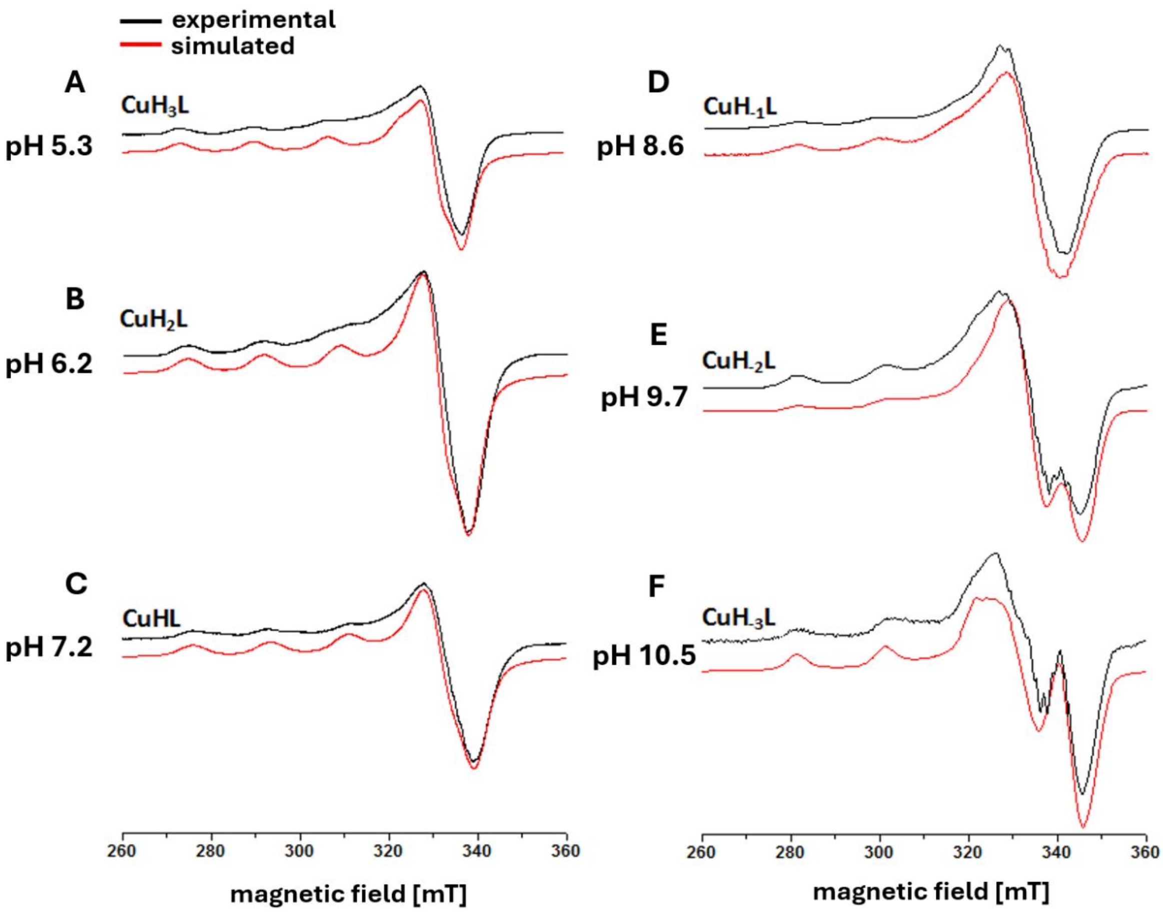

Fig.S1. EPR spectra of the frozen solution of **Cu6L** system at pH: (A) 5.3, (B) 6.2, (C) 7.2, (D) 8.6, (E) 9.7 and (F) 10.5 (Cu:L = 1:1, [Cu(II)] = 0.001 M).

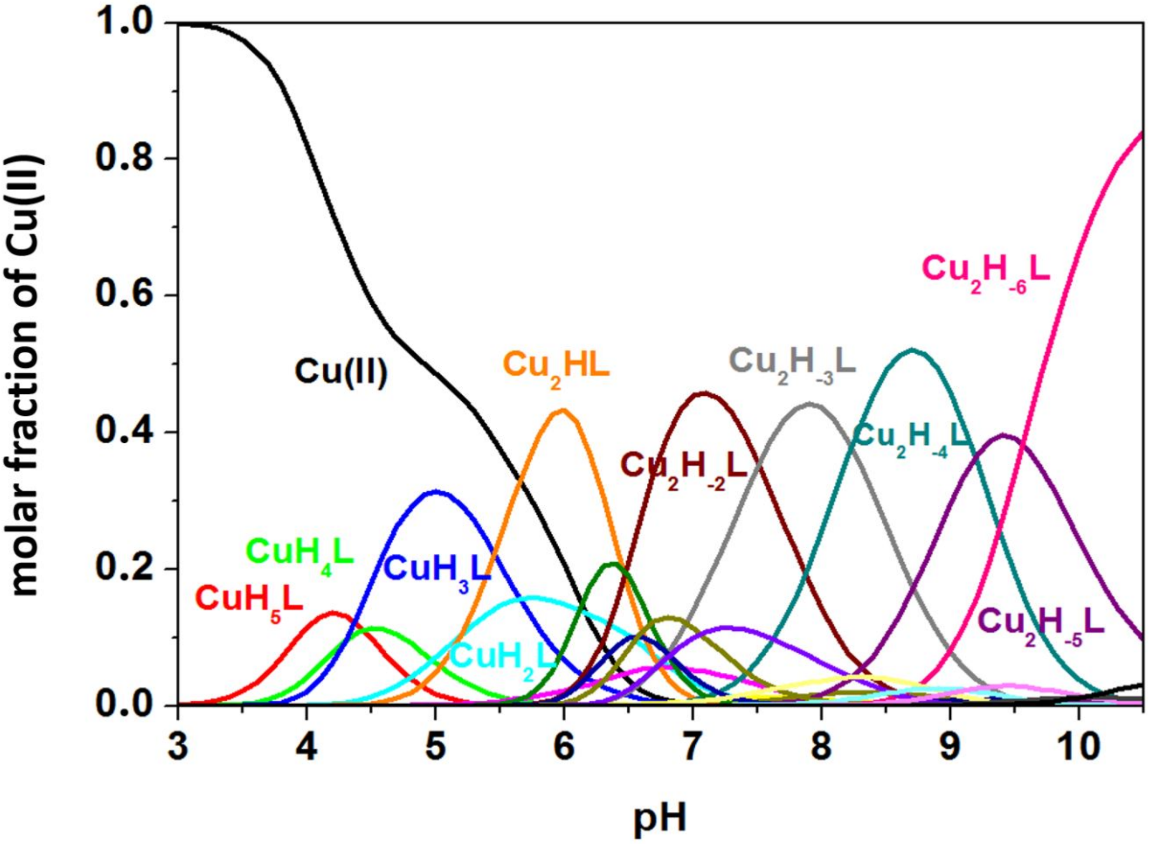

Fig.S2. Species distribution diagram of the **Cu6L** system as a function of pH. Molar ratio Cu: L = 2:1, [Cu(II)] = 0.002 M.

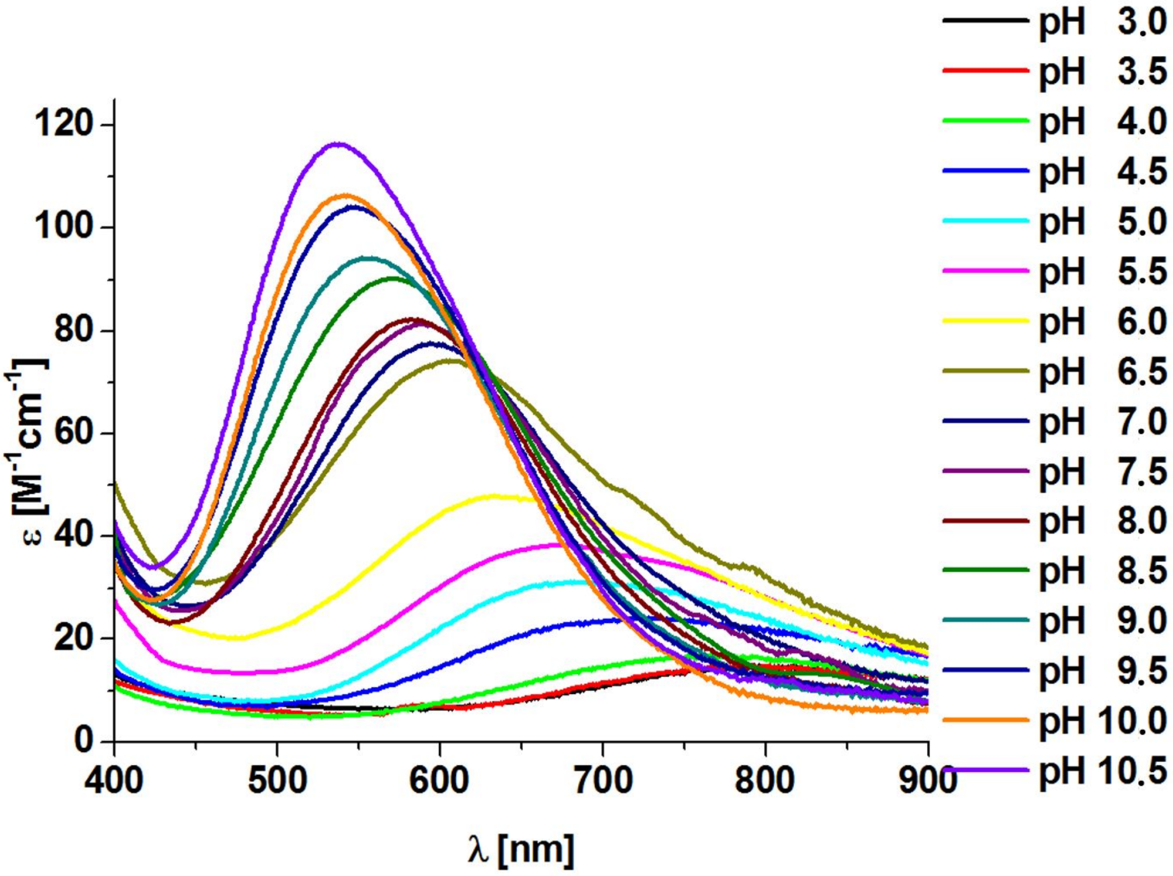

Fig.S3. Electronic absorption spectra for dinuclear complexes of the **Cu6L** system as a function of pH. Cu:L = 2:1, [Cu(II)] = 0.002 M.

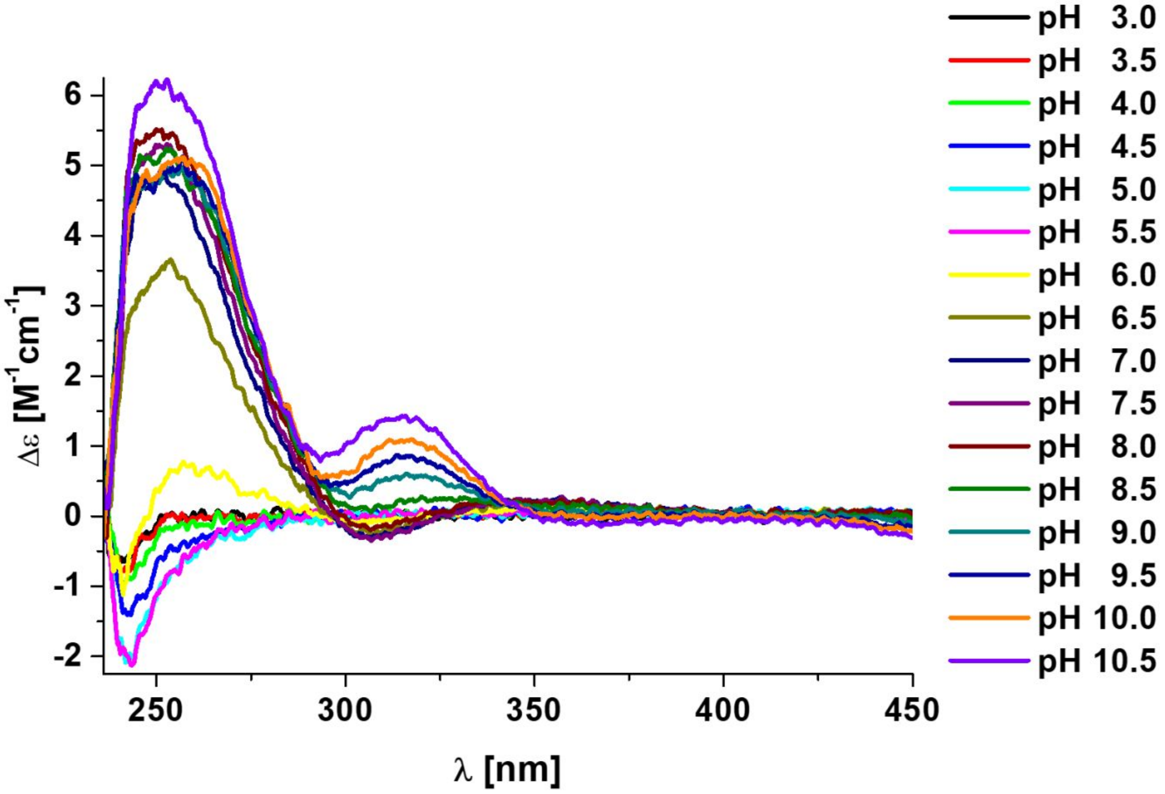

Fig.S4. CD spectra in the UV range for dinuclear complexes of the **Cu6L** system as a function of pH. Cu:L = 2:1, [Cu(II)] = 0.002 M.

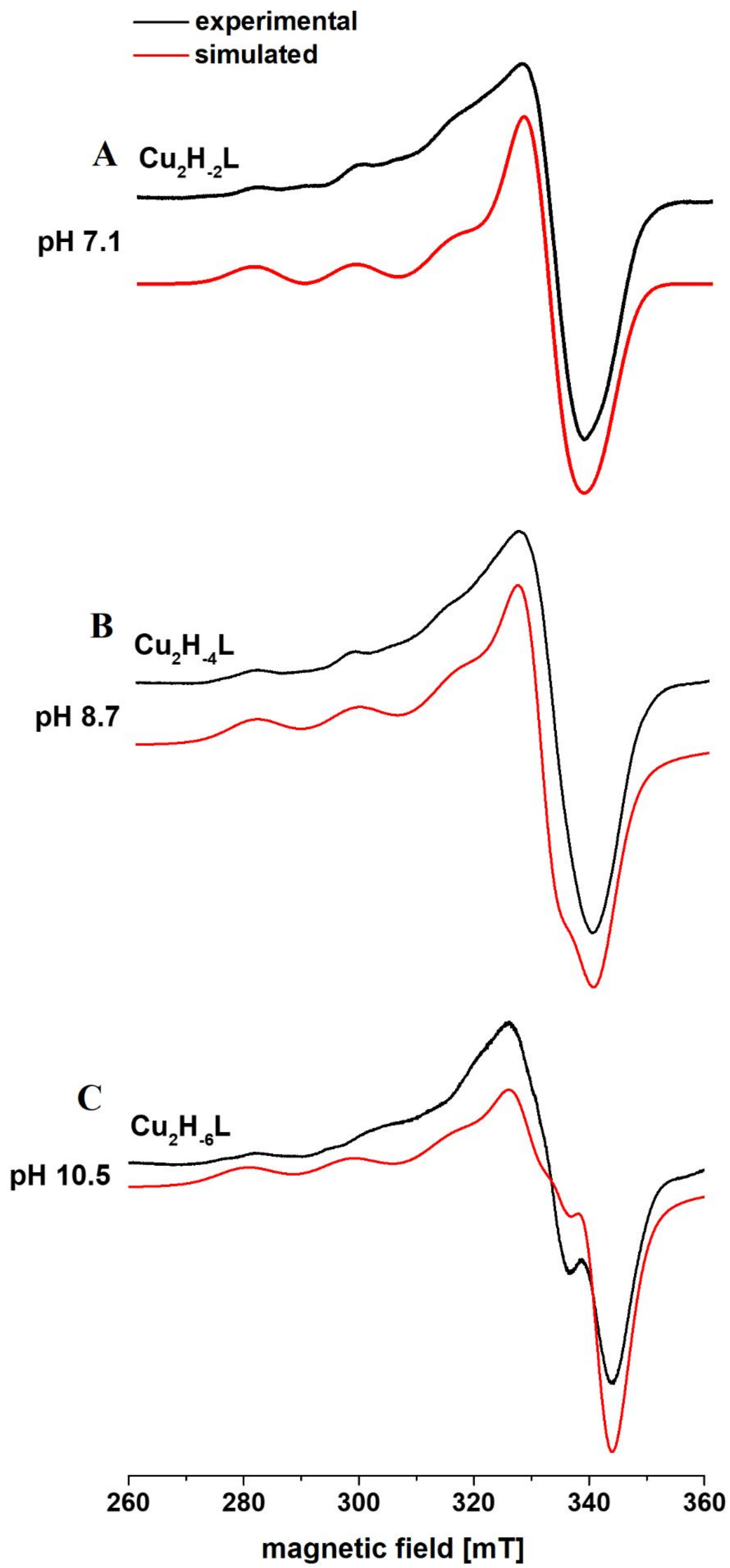

Fig.S5. EPR spectra of the frozen solution of **Cu6L** system at pH: (A) 7.1, (B) 8.7, (C) 10.5 (Cu:L = 2:1, [Cu(II)] = 0.002 M).

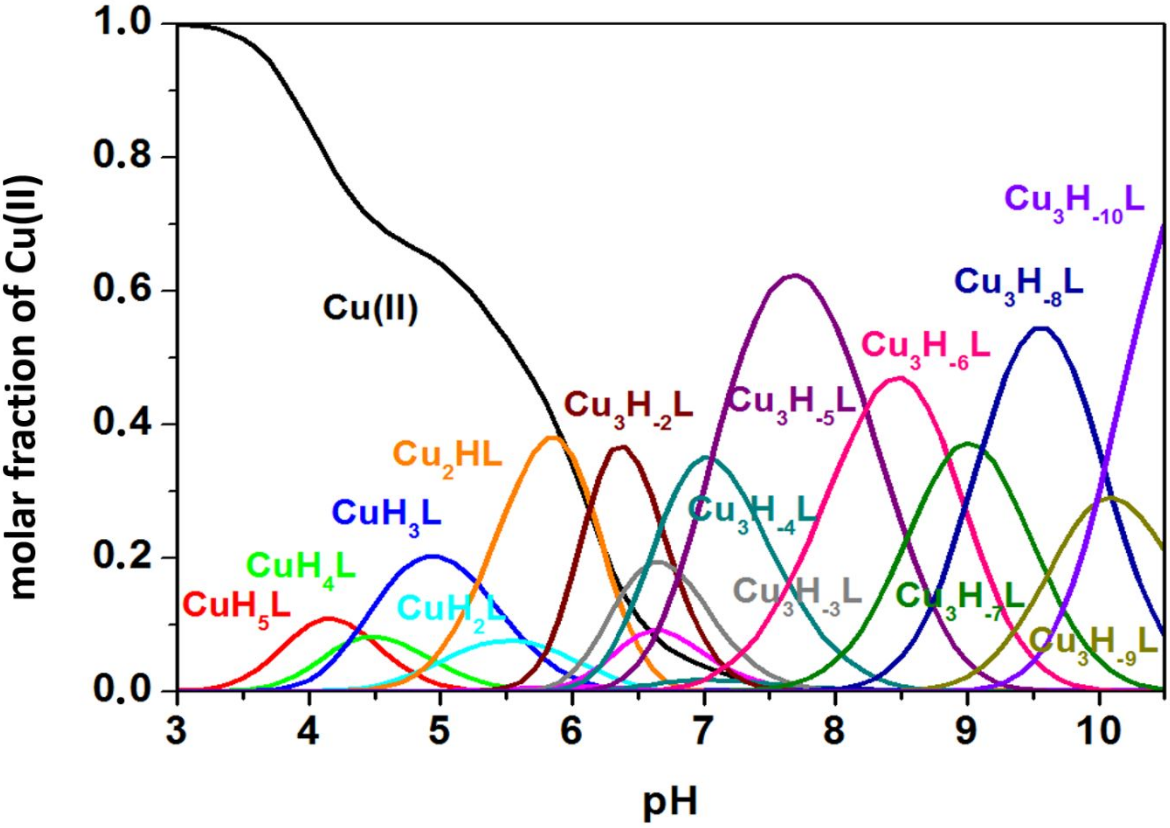

Fig.S6. Species distribution diagram of the **Cu6L** system as a function of pH. Molar ratio Cu: L = 3:1, [Cu(II)] = 0.003 M.

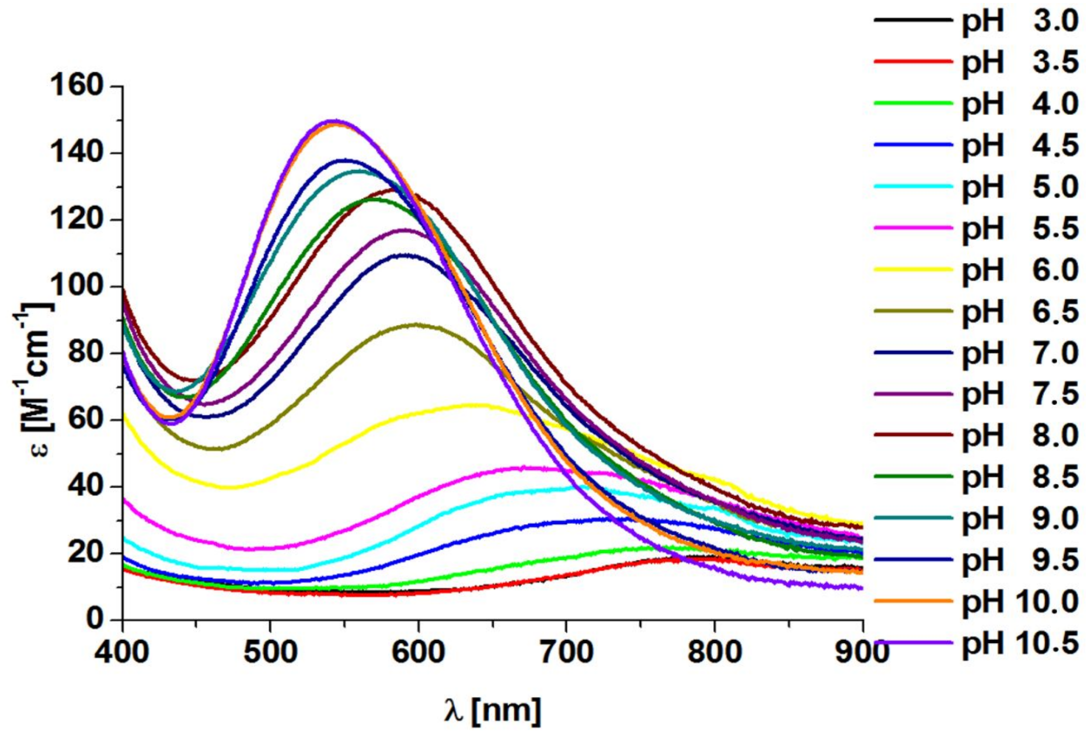

Fig.S7. Electronic absorption spectra for trinuclear complexes of the **Cu6L** system as a function of pH. Cu:L = 3:1, [Cu(II)] = 0.003 M.

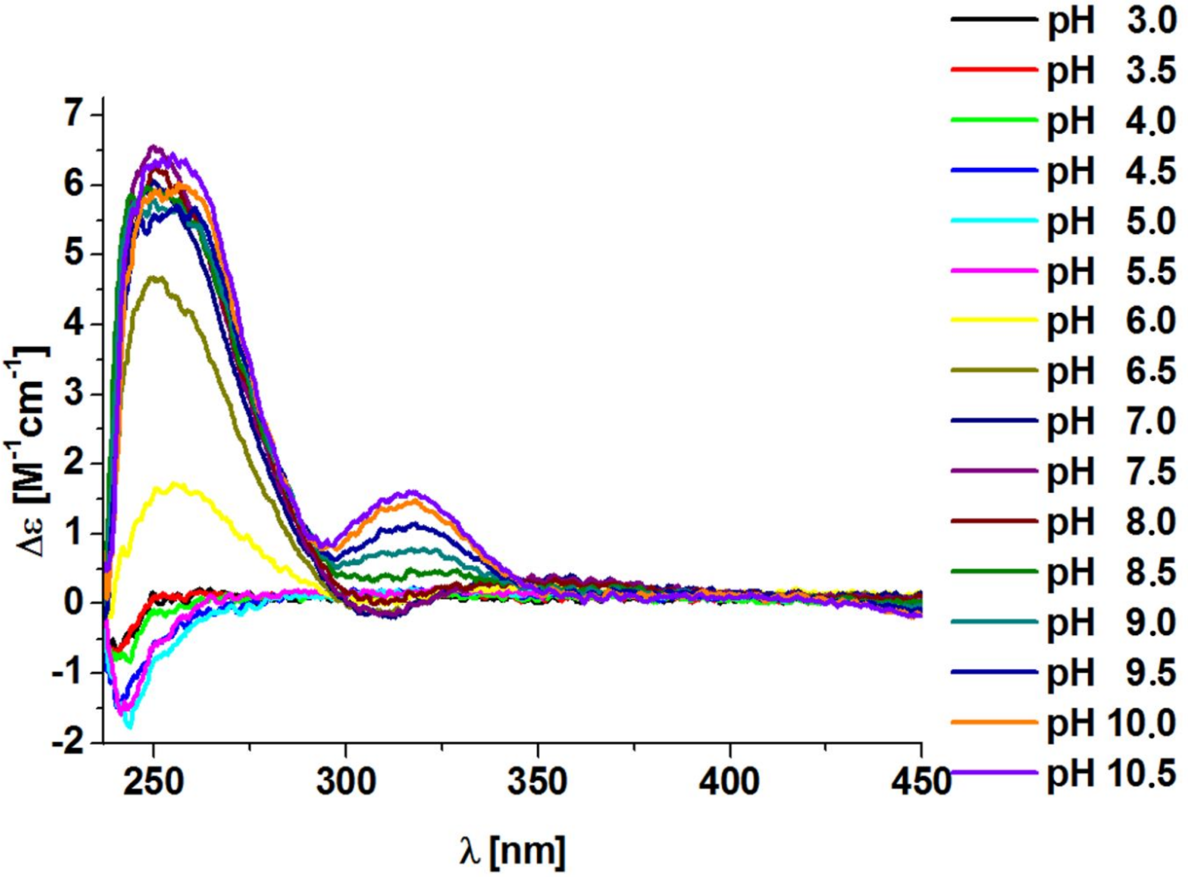

Fig.S8. CD spectra in the UV range for trinuclear complexes of the **Cu6L** system as a function of pH. Cu:L = 3:1, [Cu(II)] = 0.003 M.

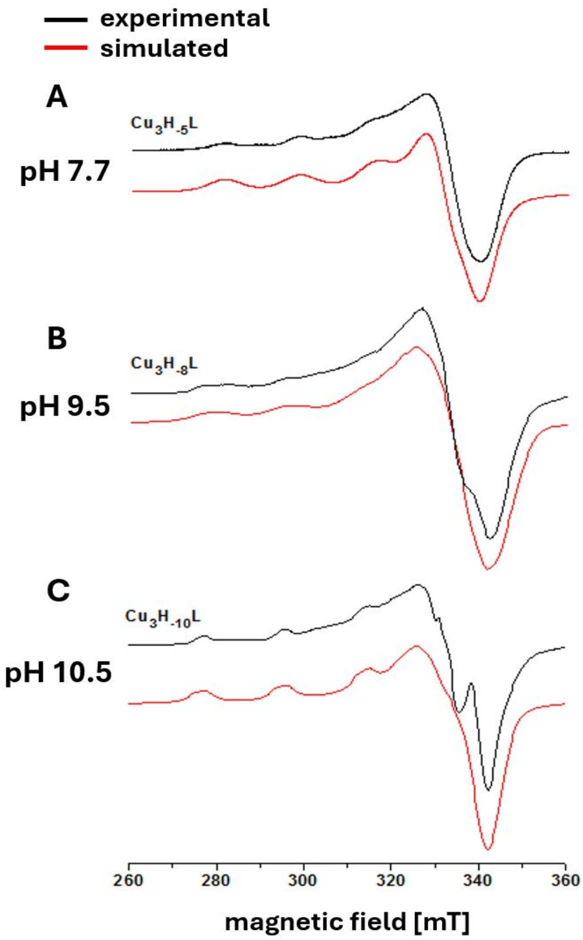

Fig.S9. EPR spectra of the frozen solution of **Cu6L** system at pH: (A) 7.7, (B) 9.5, (C) 10.5 (Cu:L = 3:1, [Cu(II)] = 0.003 M).

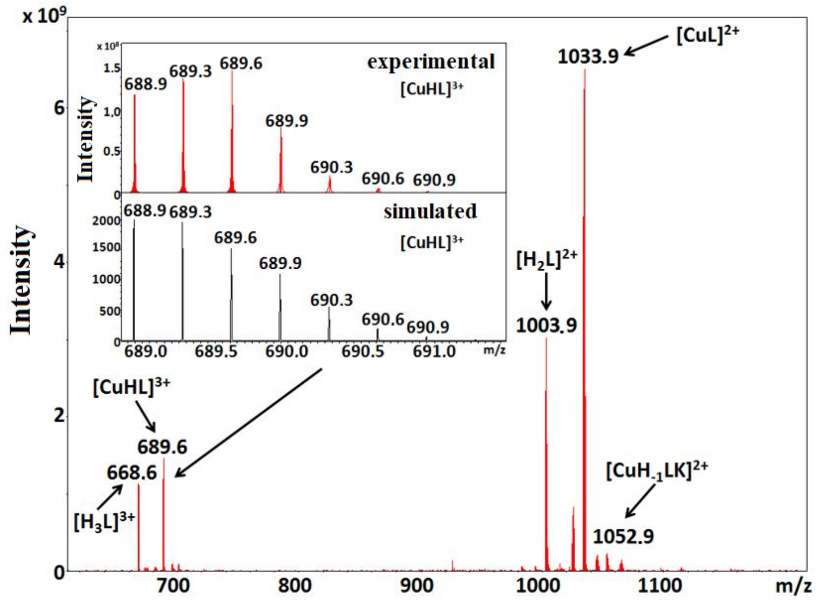

Fig.S10. ESI mass spectrum of the **Cu6L** (Cu:L = 1:1 molar ratio) in aqueous solution (pH ~ 7) along with experimental and simulated spectra of $[CuHL]^{3+}$ molecular ion (m/z = 689.6 Da).

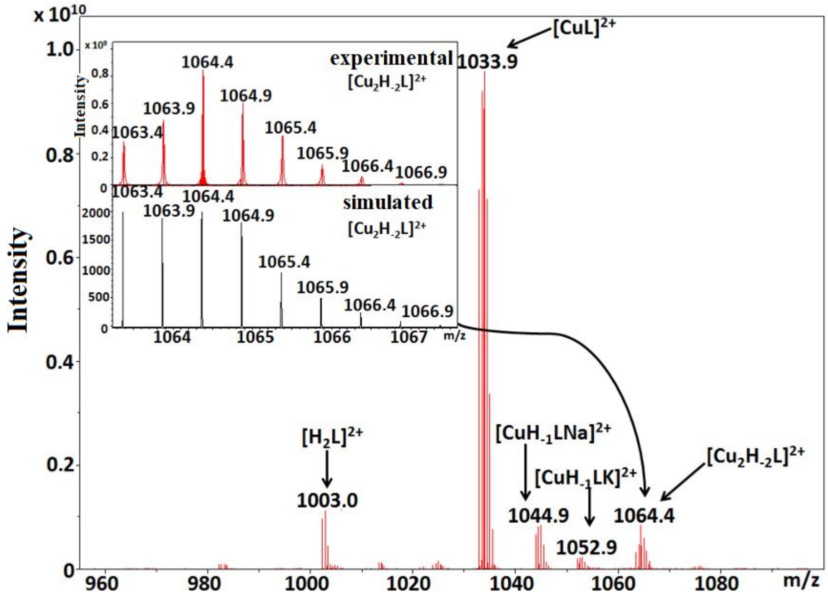

Fig.S11. ESI mass spectrum of the **Cu6L** (Cu:L = 2:1 molar ratio) in aqueous solution (pH ~ 7) along with experimental and simulated spectra of $[Cu_2H_{-2}L]^{2+}$ molecular ion (*m*/*z* = 1064.4 Da).

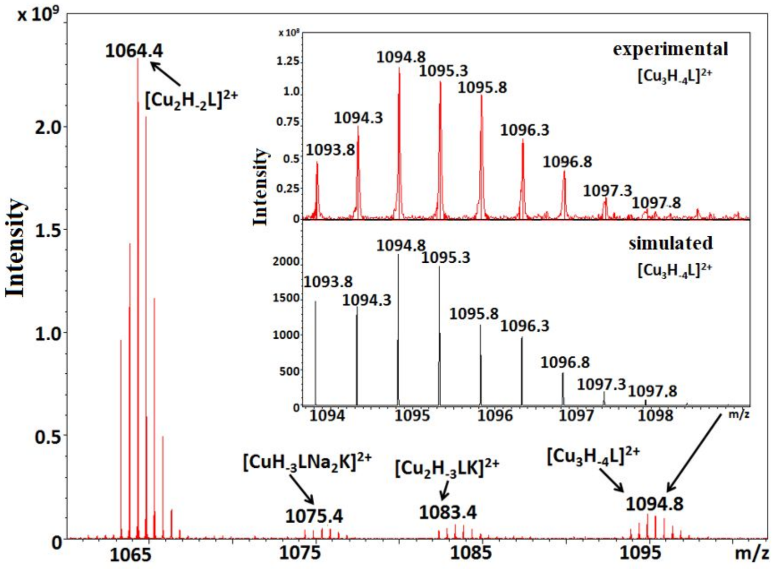

Fig.S12. ESI mass spectrum of the **Cu6L** (Cu:L = 3:1 molar ratio) in aqueous solution (pH ~ 7) along with experimental and simulated spectra of $[Cu_3H_{-4}L]^{2+}$ molecular ion (*m*/*z* = 1094.8 Da).

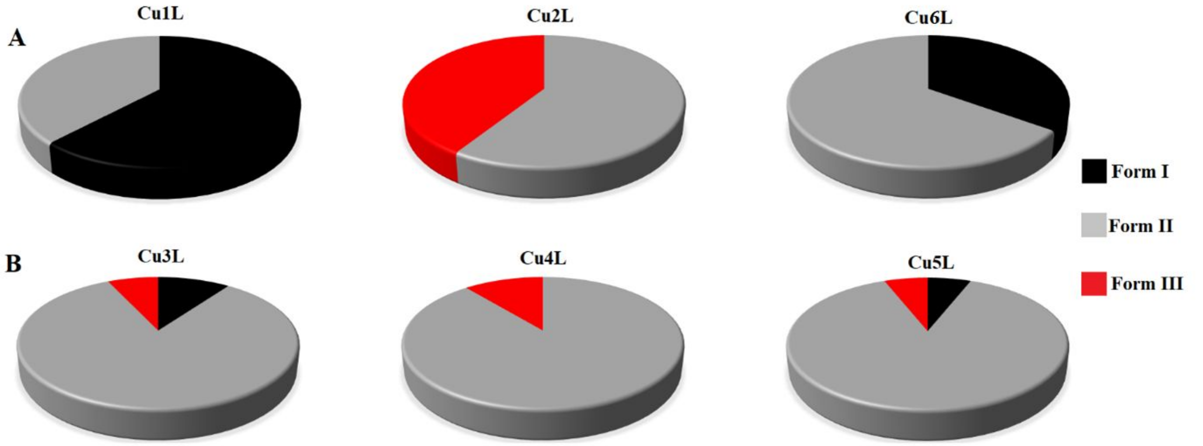

Fig.S13. Percentage of individual DNA form created after adding different Cu(II) complexes with FomA protein fragments in the presence of A) 50 µM H_2O_2 and B) 25 µM H_2O_2 to plasmid DNA.

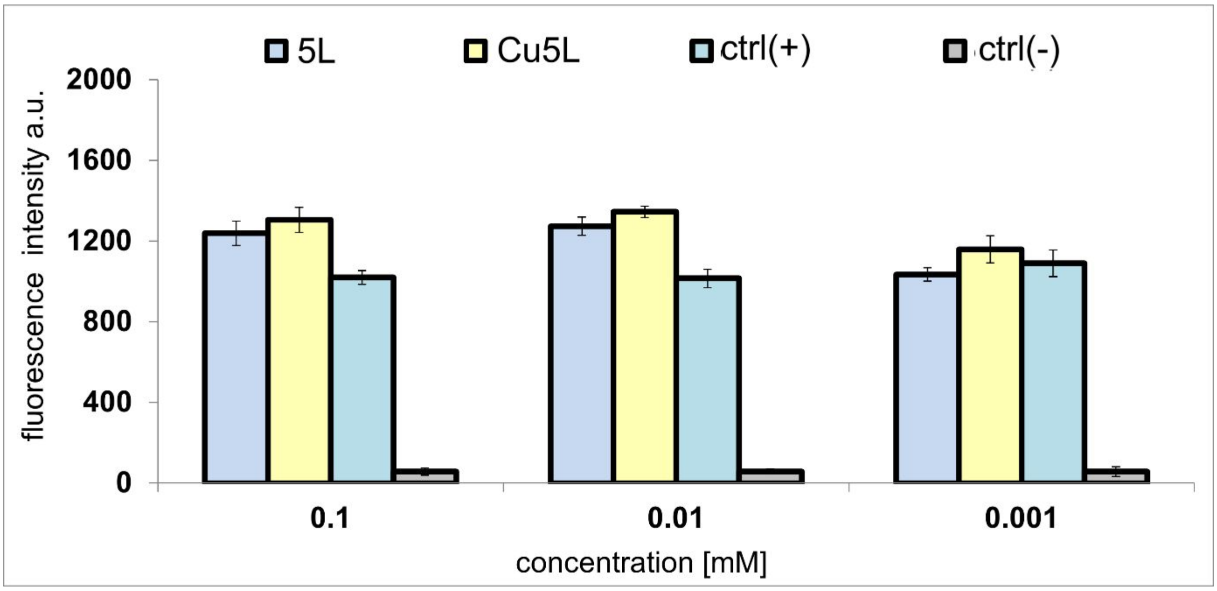

Fig.S14. Reduction of fluorescence intensity of CT26 cells incubated with **5L** and **Cu5L** at different concentrations (0.1, 0.01 and 0.001 mM) after 5 min of incubation, using H_2DCF-DA probe.

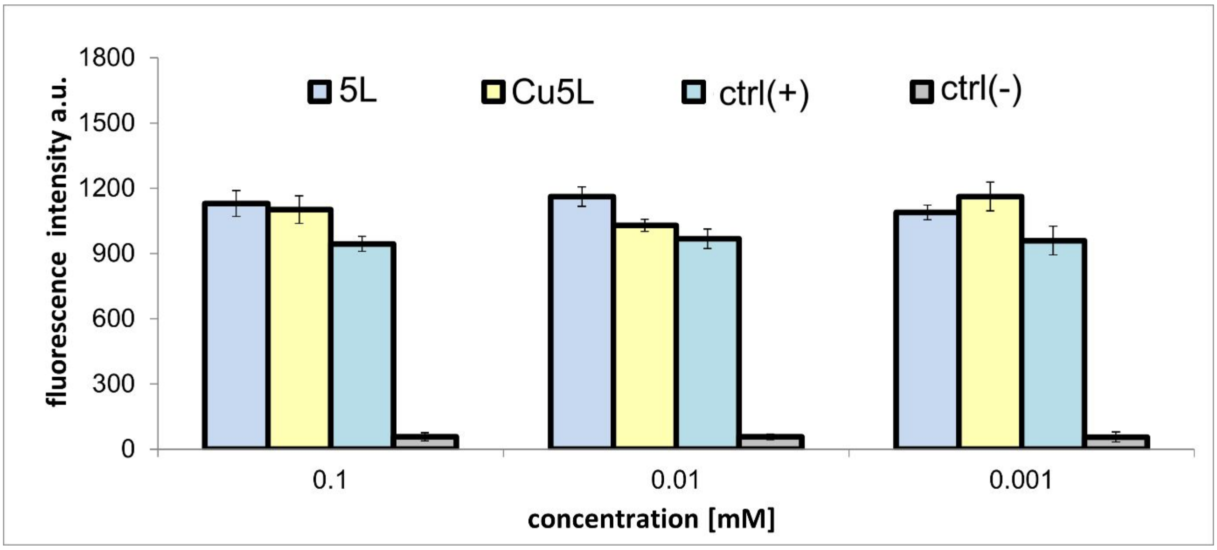

Fig.S15. Reduction of fluorescence intensity of CT26 cells incubated with **5L** and **Cu5L** at different concentrations (0.1, 0.01 and 0.001 mM) after 30 min of incubation, using H_2DCF-DA probe.

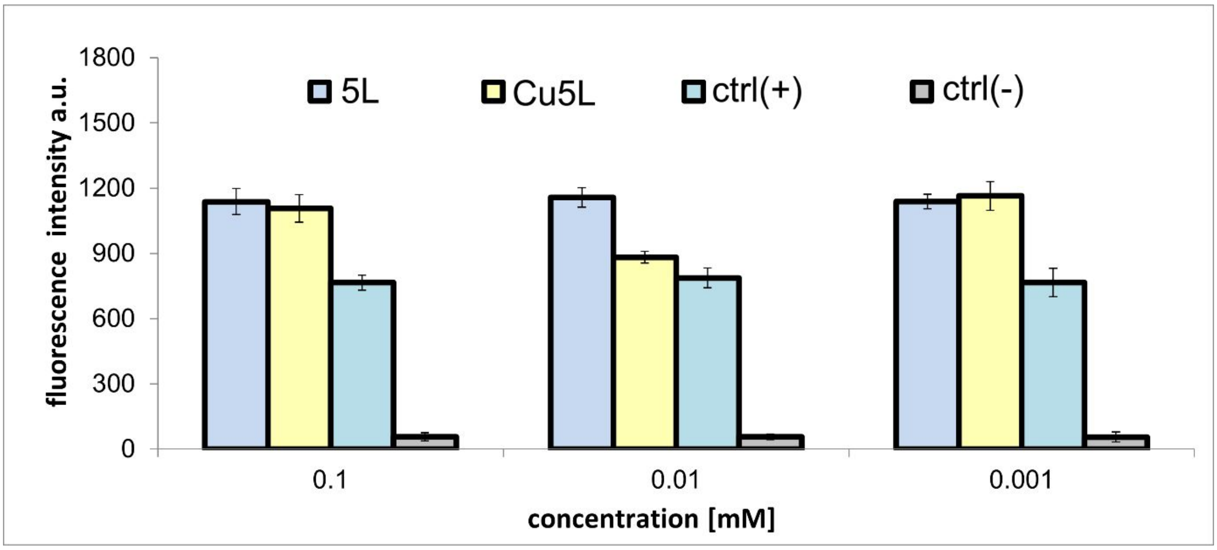

Fig.S16. Reduction of fluorescence intensity of CT26 cells incubated with **5L** and **Cu5L** at different concentrations (0.1, 0.01 and 0.001 mM) after 4 h of incubation, using H_2DCF-DA probe.

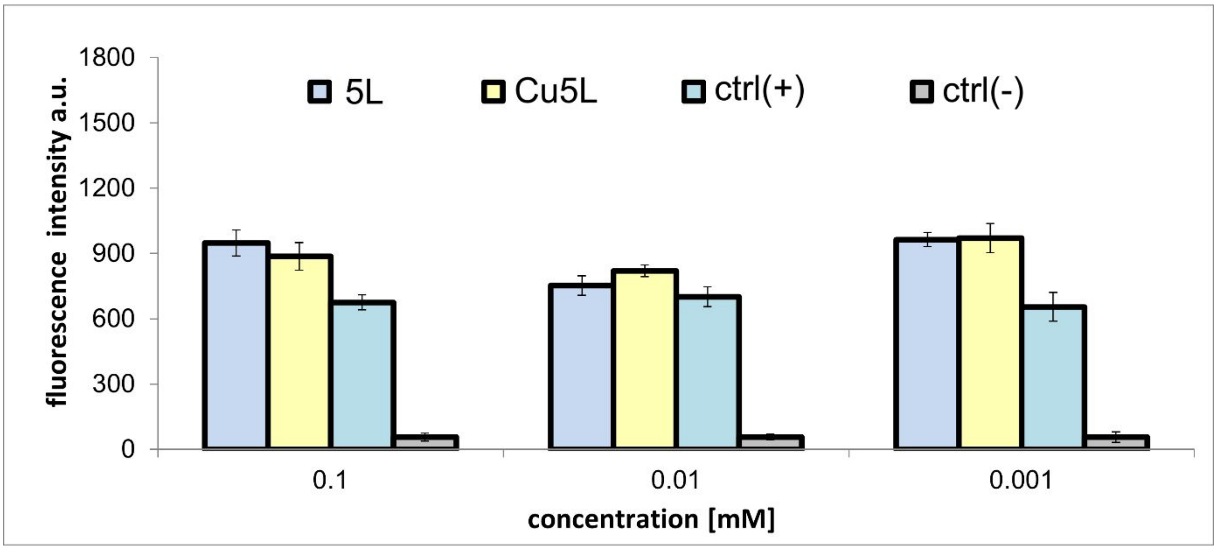

Fig.S17. Reduction of fluorescence intensity of CT26 cells incubated with **5L** and **Cu5L** at different concentrations (0.1, 0.01 and 0.001 mM) after 12 h of incubation, using H_2DCF-DA probe.

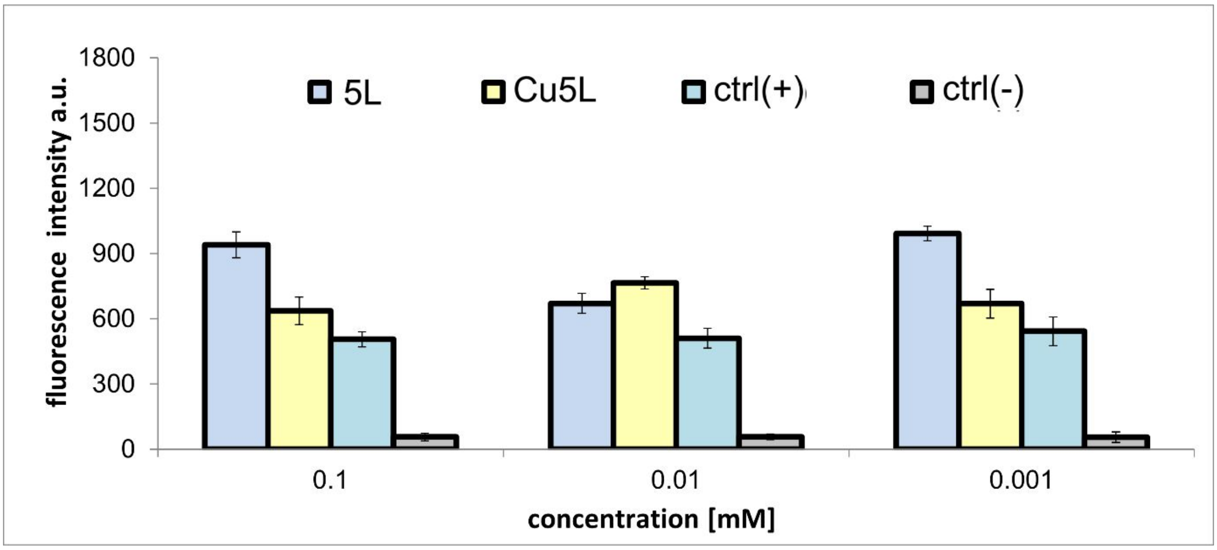

Fig.S18. Reduction of fluorescence intensity of CT26 cells incubated with **5L** and **Cu5L** at different concentrations (0.1, 0.01 and 0.001 mM) after 24 h of incubation, using H_2DCF-DA probe.

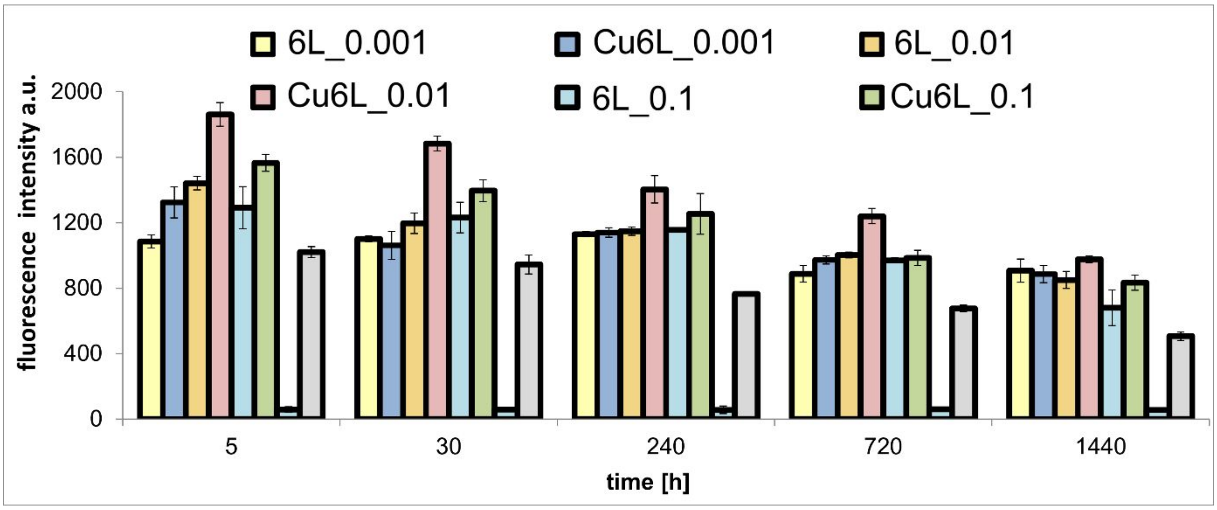

Figure S19. Reduction of fluorescence intensity of CT26 cells incubated with **6L** and **Cu6L** at increasing incubation time at different concentrations (0.1, 0.01 and 0.001mM), using H_2DCF-DA probe.

References

(1) Kowalik-Jankowska, T.; Rajewska, A.; Jankowska, E.; Grzonka, Z. Copper(II) binding by fragments of alpha-synuclein containing M1-D2- and -H50-residues; a combined potentiometric and spectroscopic study. *Dalton Trans.* **2006**, (42), 5068-5076.

(2) Kállay, C.; Várnagy, K.; Micera, G.; Sanna, D.; Sóvágó, I. Copper(II) complexes of oligopeptides containing aspartyl and glutamyl residues. Potentiometric and spectroscopic studies. *J Inorg Biochem.* **2005**, 99 (7), 1514-1525.

(3) Kowalik-Jankowska, T.; Biega, Ł.; Kuczer, M.; Konopińska, D. Mononuclear copper(II) complexes of alloferons 1 and 2: a combined potentiometric and spectroscopic studies. *J Inorg Biochem.* **2009**, 103 (1), 135-142.

(4) Kállay, C.; Nagy, Z.; Várnagy, K.; Malandrinos, G.; Hadjiliadis, N.; Sóvágó, I. Thermodynamic and structural characterization of the copper(II) complexes of peptides containing both histidyl and aspartyl residues. *Bioinorg Chem Appl.* **2007**, 2007, 30394.

(5) Błaszak, M.; Jankowska, E.; Kowalik-Jankowska, T. Copper(II) complexes of neuropeptide gamma mutant (H4A) products of metal-catalyzed oxidation. *Polyhedron*. **2014**, 68, 379-389.

(6) Puntervoll, P,; Ruud, M.; Bruseth, L. J.; Kleivdal, H.; Høgh, B. T.; Benz, R.; Jensen, H. B. Structural characterization of the fusobacterial non-specific porin FomA suggests a 14-stranded topology, unlike the classical porins. *Microbiology* **2002**, 148, (Pt 11), 3395-3403.

(7) Liu, P. F.; Shi, W.; Zhu, W.; Smith, J. W.; Hsieh, S. L.; Gallo, R. L.; Huang, C. M. Vaccination targeting surface FomA of Fusobacterium nucleatum against bacterial co-aggregation: Implication for treatment of periodontal infection and halitosis. *Vaccine.* **2010**, 28 (19), 3496-3505.

(8) Nugent, S. G.; Kumar, D.; Rampton, D. S.; Evans, D. F. Intestinal luminal pH in inflammatory bowel disease: possible determinants and implications for therapy with aminosalicylates and other drugs. *Gut.* **2001**, 48 (4), 571-577.

(9) Kowalik-Jankowska, T.; Lesiów, M.; Krupa, K.; Kuczer, M.; Czarniewska, E. Copper(ii) complexes with alloferon analogues containing phenylalanine H6F and H12F stability and biological activity lower stabilization of complexes compared to analogues containing tryptophan. *Metallomics*. **2019**, 11 (10), 1700-1715.

(10) Kowalik-Jankowska, T.; Ruta-Dolejsz, M.; Wiśniewska, K.; Lankiewicz, L. Coordination of copper(II) ions by the 11-20 and 11-28 fragments of human and mouse beta-amyloid peptide. *J Inorg Biochem.* **2002**, 92 (1), 1-10.

(11) Zieliński, W.; Rajca, A. Metody spektroskopowe i ich zastosowanie do identyfikacji związków organicznych, *Wydawnictwo Naukowo-Techniczne*. Warszawa, **1995**.

(12) Arus, D.; Jancsó, A.; Szunyogh, D.; Matyuska, F.; Nagy, N. V.; Hoffmann, E.; Körtvélyesi, T.; Gajda, T. On the possible roles of N-terminal His-rich domains of Cu,Zn SODs of some Gram-negative bacteria. *J Inorg Biochem.* **2012**, 106 (1), 10-18.

(13) De Angelis, F.; Lee, J. K.; O'Connell 3rd, J. D.; Miercke, L. J.; Verschueren, K. H.; Srinivasan, V.; Bauvois, C.; Govaerts, C.; Robbins, R. A.; Ruysschaert, J. M.; Stroud, R. M.; Vandenbussche, G. Metal-induced conformational changes in ZneB suggest an active role of

membrane fusion proteins in efflux resistance systems. *Proc Natl Acad Sci U S A.* **2010**, 107 (24), 11038-11043.

(14) Kowalik-Jankowska, T.; Kadej, A.; Kuczer, M.; Czarniewska, E. Copper(II) complexes of the Neb-colloostatin analogues containing histidine residue structure stability biological activity. *Polyhedron*. **2017**, 134, 365-375.

(15) Brasuń J.; Matera, A.; Ołdziej, S.; Swiatek-Kozłowska, J.; Messori, L.; Gabbiani, C.; Orfei, M.; Ginanneschi, M. The copper(II) coordination abilities of three novel cyclic tetrapeptides with -His-Xaa-His- motif. *J Inorg Biochem.* **2007**, 101 (3), 452-460.

(16) Lesiów, M. K.; Bieńko, A.; Sobańska, K.; Kowalik-Jankowska, T.; Rolka, K.; Łęgowska, A.; Ptaszyńska, N. Cu(II) complexes with peptides from FomA protein containing -His-Xaa-Yaa-Zaa-His and -His-His-motifs. ROS generation and DNA degradation. *J Inorg Biochem.* **2020**, 212, 111250.

(17) Lesiów, M. K.; Komarnicka, U. K.; Stokowa-Sołtys, K.; Rolka, K.; Łęgowska, A.; Ptaszyńska, N.; Wieczorek, R.; Kyzioł, A.; Jeżowska-Bojczuk, M. Relationship between copper(ii) complexes with FomA adhesin fragments of F. nucleatum and colorectal cancer. Coordination pattern and ability to promote ROS production. *Dalton Trans.* **2018**, 47 (15), 5445-5458.

(18) Kowalik-Jankowska, T.; Jankowska, E.; Szewczuk, Z.; Kasprzykowski, F. Coordination abilities of neurokinin A and its derivative and products of metal-catalyzed oxidation. *J Inorg Biochem.* **2010**, 104 (8), 831-842.

(19) Kowalik-Jankowska, T.; Ruta-Dolejsz, M.; Wisniewska, K.; Lankiewicz, L.; Kozlowski, H. Copper(II) complexation by human and mouse fragments (11-16) of ß-amyloid peptide. *J. Chem. Soc. Dalt. Trans.* **2000**, (24), 4511-4519.

(20) Prenesti, E.; Daniele, P. G.; Prencipe, M.; Ostacoli, G. Spectrum-structure correlation for visible absorption spectra of copper(II) complexes in aqueous solution. *Polyhedron*. **1999**, 18 (25), 3233-3241.

(21) Brasuń, J.; Matera-Witkiewicz, A.; Ołdziej, S.; Pratesi, A.; Ginanneschi, M.; Messori, L. Impact of ring size on the copper(II) coordination abilities of cyclic tetrapeptides. *J Inorg Biochem.* **2009**, 103 (5), 813-817.

(22) Panagiotou, K.; Panagopoulou, M.; Karavelas, T.; Dokorou, V.; Hagarman, A.; Soffer, J.; Schweitzer-Stenner, R.; Malandrinos, G.; Hadjiliadis, N. Cu(II) and Ni(II) interactions with the terminally blocked hexapeptide Ac-Leu-Ala-His-Tyr-Asn-Lys-amide model of histone H2B (80-85). *Bioinorg Chem Appl.* **2008**, 2008, 257038.

(23) Kotynia, A.; Bielińska, S.; Kamysz, W.; Brasuń, J. The coordination abilities of the multiHis-cyclopeptide with two metal-binding centers--potentiometric and spectroscopic investigation. *Dalton Trans.* **2012**, 41 (39), 12114-12120.

(24) Kadej, A.; Kuczer, M.; Kowalik-Jankowska, T. Copper(II) complexes of terminally free alloferon mutants containing two histidyl binding sites inside peptide chain structure and stability. *Dalton Trans.* **2015**, 44 (47), 20659-20674.

(25) Matusiak, A.; Kuczer, M.; Czarniewska, E.; Urbański, A.; Rosiński, G.; Kowalik-Jankowska, T. Copper(II) complexes of terminally free alloferon peptide mutants containing two different histidyl (H(1) and H(6) or H(9) or H(12)) binding sites Structure Stability and Biological Activity. *J Inorg Biochem.* **2015**, 151, 44-57.

(26) Vázquez-Salazar, A.; Becerra, A.; Lazcano, A. Evolutionary convergence in the biosyntheses of the imidazole moieties of histidine and purines. *PLoS One.* **2018**, 13 (4), e0196349.

(27) Kuczer, M.; Błaszak, M.; Czarniewska, E.; Rosiński, G.; Kowalik-Jankowska, T. Mono- and polynuclear copper(II) complexes of alloferons 1 with point mutations (H6A) and (H12A): stability structure and cytotoxicity. *Inorg Chem.* **2013**, 52 (10), 5951-5961.
